# Deep Eutectic Solvents Ignition-Triggered Interfacial Fusion for Structural-Grade Lignocellulosic Boards

**DOI:** 10.34133/research.1365

**Published:** 2026-07-17

**Authors:** Huiru Yue, Cheng Zuo, Xinyi Hui, Jia-Long Wen, Tong-Qi Yuan

**Affiliations:** ^1^ State Key Laboratory of Efficient Production of Forest Resources, Beijing Key Laboratory of Lignocellulosic Chemistry, Beijing Forestry University, Beijing 100083, China.; ^2^ Hebei Key Laboratory of Agricultural and Forestry Biomass Materials Science and Application, Beijing Forestry University, Xiong’an 070001, China.

## Abstract

Lignocellulosic structural boards are often regarded as sustainable materials, yet most still depend on fossil-derived thermoset resins that generate volatile emissions, form weak and irreversible bonded interfaces, and complicate circular end-of-life management. Here, we introduce deep eutectic solvent (DES) ignition-triggered interfacial fusion, a closed-loop strategy that replaces external adhesives through a 2-stage sequence. In the ignition stage, a minimal amount of recyclable DES selectively activates lignocellulosic cell walls and mobilizes native lignin/hemicellulose toward particle contact zones, creating an interface-ready state. In the subsequent fusion stage, hot pressing reconstructs and locks these redistributed components into a continuous, lignin-rich bonding interphase, yielding an adhesive-free BioFuse-Board. The resulting BioFuse-Board delivers an internal bond strength up to 2.53 MPa (vs. the 0.45 MPa commercial requirement) and a 24-h thickness swelling of 10.05%. Mechanistically, ignition promotes β–*O*–4 bond cleavage, lowers lignin molecular weight, and enriches phenolic and carboxyl sites, whereas fusion promotes condensation while depleting reactive sites, consistent with a locked interfacial network involving oxygen-bridged environments, lignin-rich C–C connectivity, and strengthened hydrogen bonding with cellulose. Beyond performance, the same ignition–fusion architecture embeds circularity via reagent reuse, stream valorization into wood adhesives, and mechanical remanufacturing of end-of-life boards. Technoeconomic and cradle-to-gate life-cycle analyses further demonstrate that DES ignition-triggered interfacial fusion is economically viable and substantially reduces environmental impacts. Collectively, this strategy provides a generalizable route to circular manufacturing of structural-grade, adhesive-free lignocellulosic structural materials.

## Introduction

**Fig. 1.**
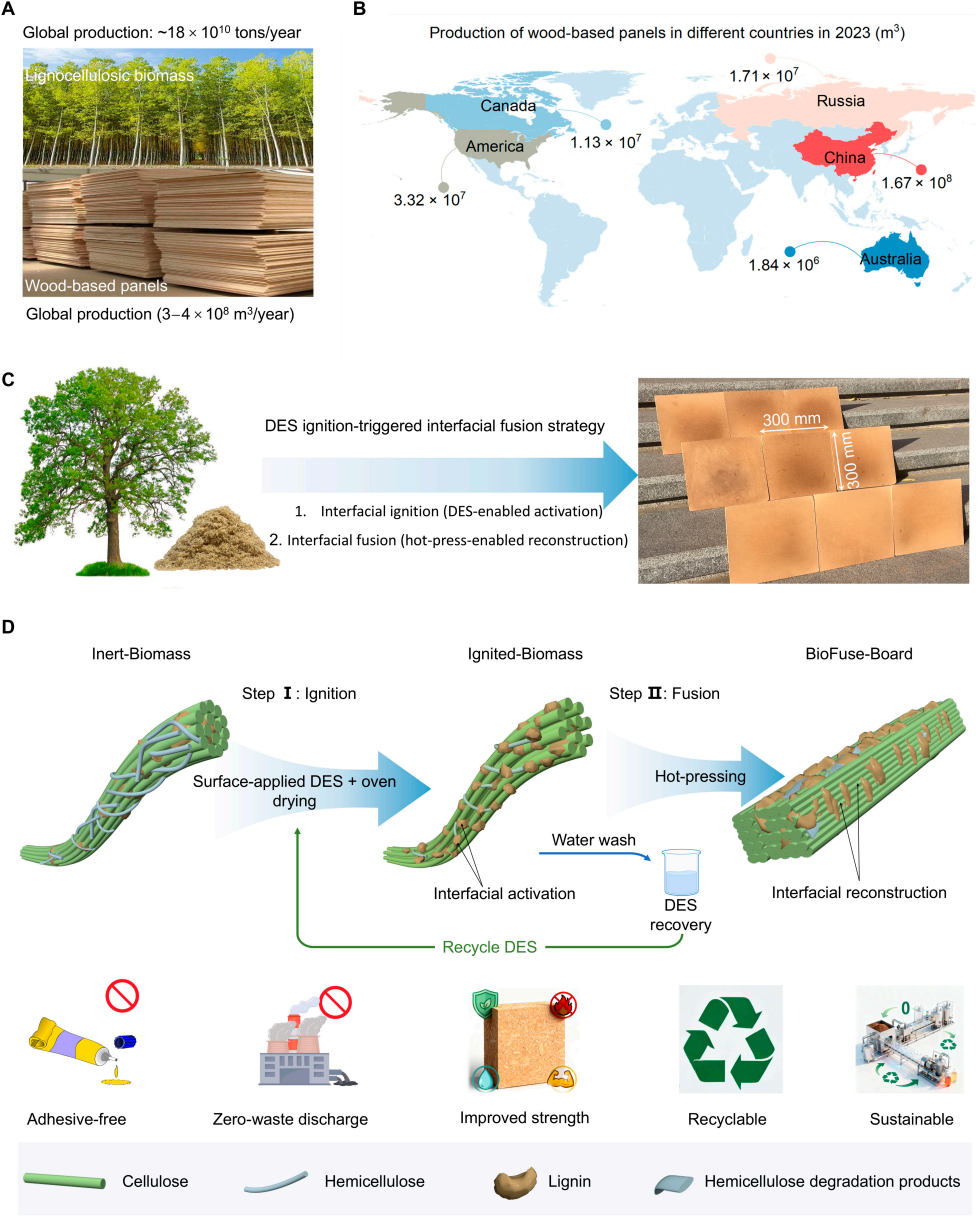
Deep eutectic solvent (DES) ignition-triggered interfacial fusion for closed-loop lignocellulosic structural boards. (A) Global annual availability of lignocellulosic biomass and the large production scale of wood-based panels. (B) Geographic distribution of wood-based panel production (2023), highlighting the scale and regional concentration of the industry. (C and D) Concept schematic of DES ignition-triggered interfacial fusion: Inert-Biomass is transformed into BioFuse-Board through DES-enabled interfacial ignition (interface activation) followed by DES removal/recovery and hot-pressing fusion (interface reconstruction). After ignition, residual DES is removed by water washing, recovered from the filtrate, and recycled before hot pressing. Target attributes enabled by this strategy include adhesive-free manufacturing, reduced discharge through solvent reuse, improved structural performance, recyclability, and sustainability.

Structural materials are essential to engineering sectors such as construction, automotive manufacturing, and aerospace [1–4]. However, their production still relies heavily on fossil-derived inputs and is increasingly constrained by carbon-neutrality targets [5–7]. Nature provides approximately 180 billion tons of lignocellulosic biomass annually, making lignocellulosic biocomposites a viable renewable alternative to petroleum-based composites and traditional structural materials (Fig. 1A) [8–11]. Among these materials, wood-based panels represent one of the largest industrial outlets for lignocellulosic feedstocks, with an annual production scale of approximately 300 to 400 million tons and a geographically concentrated manufacturing footprint (Fig. 1B) [12–14]. However, most wood-based panels still depend on synthetic thermoset adhesives, including urea–formaldehyde, melamine–urea–formaldehyde, phenol–formaldehyde, and isocyanate resins, to meet mechanical and durability requirements. These fossil-derived binders increase cost and processing complexity and, more importantly, release regulated volatiles such as formaldehyde, raising serious environmental and health concerns [15,16].

To mitigate these issues, bio-based adhesives derived from polysaccharides, proteins, lignin, or tannins have been developed [17–21]. Although such alternatives offer renewability and low or zero formaldehyde emissions, they often require blending with conventional resins to achieve satisfactory performance [22]. In parallel, adhesive-free self-bonding strategies have been explored, including oxidation bonding, radical initiation, acid or alkaline activation, natural-substance conversion, and enzymatic approaches [23–26]. These methods often improve cohesion at the expense of process simplicity, controllability, or reversibility. Many rely on strong acids or alkalis, oxidants, or radical initiators, yet these chemistries are often difficult to control at the cell wall scale and are rarely designed with reagent recovery or remanufacturing in mind [27–30]. Adding isolated lignin increases solids handling and often reintroduces a binder-like supply chain, whereas extracting high-purity lignin is time-consuming, reagent-intensive, and waste-generating [31]. The central limitation is therefore not whether lignin can be modified but whether interfacial cohesion can be programmed from native components under conditions compatible with dry pressing and circular operation. Under industrial hot pressing, however, both adhesive substitution and self-bonding approaches still face practical trade-offs. Gains in performance frequently rely on complex chemistry or substantial chemical inputs, and they often compromise recyclability or manufacturing compatibility.

A key opportunity is to unlock the native “adhesive” potential of lignin and hemicellulose without introducing an external binder. Deep eutectic solvents (DESs) offer an attractive green-solvent platform for lignocellulosic interfacial activation because they can be prepared from hydrogen bond acceptor/donor pairs, possess low vapor pressure, are relatively easy to synthesize, and can be structurally tuned to interact with lignin and hemicellulose through hydrogen bonding, Lewis acid coordination, and solvation effects [32–36]. Importantly, the practical sustainability of DES-based processing depends on solvent composition, dosage, recovery efficiency, and reuse stability. Therefore, using DES as a recoverable interfacial activation reagent, rather than as a large-volume bulk pretreatment medium, provides a more suitable route for greener lignocellulosic board manufacturing. Yet most DES-enabled lignocellulosic routes still deploy DES primarily as a liquid medium for pretreatment, where performance depends on maintaining a large solvent inventory and extraction process [37–39]. This paradigm is difficult to reconcile with dry particulate feedstocks and continuous hot pressing, and it rarely treats solvent circulation as a first-class design variable for circular manufacturing. The field therefore still lacks a DES strategy that can operate with a low solvent inventory while remaining compatible with closed-loop operation, dry pressing, and board remanufacturing. Here, low solvent inventory refers to using only a limited amount of DES relative to biomass, so that the solvent functions as a surface-applied interfacial activation reagent rather than as a bulk liquid medium for full biomass solvation or fractionation. This distinction is important because conventional DES pretreatment typically relies on immersing biomass in a large solvent phase, which increases solvent handling and recovery burdens and is difficult to integrate with dry particulate board manufacturing. This gap motivates the key scientific question addressed here: Can a small and recoverable DES inventory locally ignite lignin/hemicellulose redistribution at cell wall interfaces during thermal treatment and, upon hot pressing, fuse and lock these native components into a continuous load-bearing interphase?

To address this challenge, we propose DES ignition-triggered interfacial fusion as a closed-loop manufacturing strategy for structural-grade lignocellulosic boards (Fig. 1C). In this strategy, loose lignocellulosic particles are converted into adhesive-free BioFuse-Boards through a 2-step interfacial programming process. DES treatment first “ignites” the particle interfaces by selectively activating lignocellulosic cell walls and mobilizing native lignin/hemicellulose toward particle contact zones, creating an interface-ready precursor state. Subsequent hot pressing then “fuses” these ignited interfaces by consolidating redistributed components into a continuous, lignin-rich bonding interphase without external adhesives (Fig. 1D). This route delivers a high internal bond (*IB*) strength of 2.53 MPa (versus the 0.45-MPa commercial requirement) together with excellent dimensional stability, with 24-h thickness swelling of 10.05%. Importantly, DES ignition-triggered interfacial fusion strategy closes material loops at multiple levels. The DES can be recovered and reused across multiple cycles while retaining useful bonding performance and strong water stability within a bounded operating window. Spent solvent streams can be repurposed into wood adhesives to fabricate high-performance plywood, and end-of-life boards can be mechanically remanufactured into regenerated products (BioFuse-Board-R1, *IB* 2.55 MPa and thickness swelling 7.00%). Technoeconomic analysis (TEA) and cradle-to-gate life cycle assessment (LCA) further support the economic viability ad reduced environmental impacts of this closed-loop strategy.

## Results and Discussion

### DES ignition-triggered interfacial fusion defines structural-grade processing window for BioFuse-Boards

**Fig. 2.**
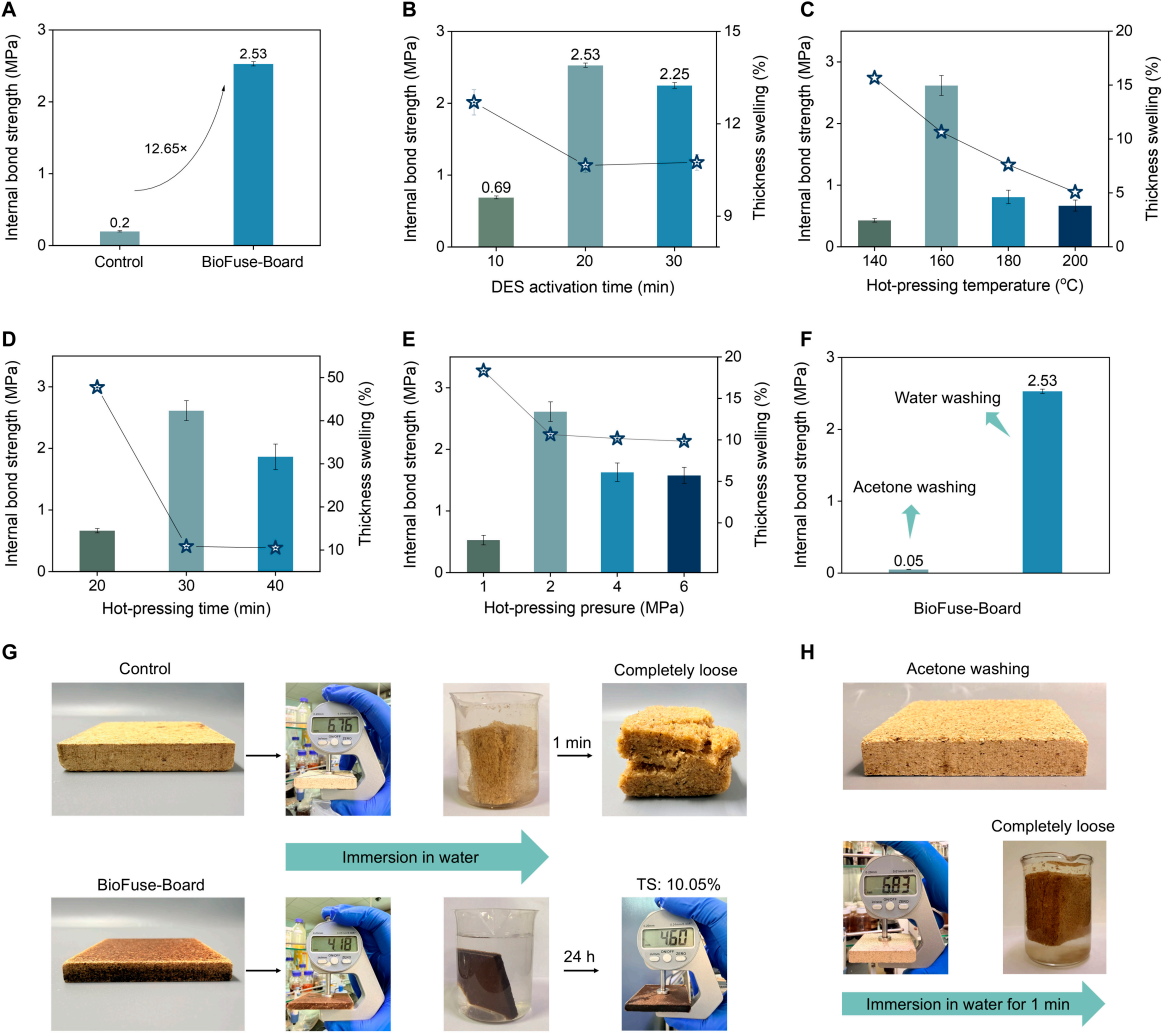
Structural-grade processing window defined by deep eutectic solvent (DES) ignition and hot-pressing fusion. (A) Internal bond (IB) strength comparison between boards made by direct hot pressing of untreated poplar particles (control) and BioFuse-Board produced via DES ignition followed by hot-pressing fusion. (B) Effect of DES ignition time on *IB* strength (bars) and 24-h thickness swelling (line), identifying an optimal ignition duration. (C) Effect of hot-pressing temperature on *IB* strength (bars) and thickness swelling (line) at fixed 2 MPa and 30 min. (D) Effect of hot-pressing time on *IB* strength (bars) and thickness swelling (line) at fixed 160 °C and 2 MPa. (E) Effect of hot-pressing pressure on *IB* strength (bars) and thickness swelling (line) at fixed 160 °C and 30 min. (F) *IB* strength of BioFuse-Boards prepared from Ignited-Biomass after water washing or acetone washing before hot pressing. (G) Water-immersion resistance comparison between the control and BioFuse-Board, showing rapid disintegration of the control and retained integrity of BioFuse-Board after soaking (with thickness swelling [*TS*] annotated). (H) Water-immersion behavior of the acetone-washed board, showing rapid disintegration within 1 min, similar to the directly hot-pressed untreated wood-powder control.

We first mapped the processing window in which DES-enabled interfacial ignition and fusion convert loose poplar particles into a structural-grade, adhesive-free board. Interfacial activation is central to this performance (Figs. S1 and S2). Figure 2A compares BioFuse-Board produced by direct hot pressing of natural poplar particles with boards prepared after DES ignition followed by hot-press consolidation. Direct hot pressing of untreated substrate yielded an IB strength of only 0.20 MPa. In contrast, boards prepared by 20-min oven activation at 120 °C under identical hot-pressing conditions exhibited an *IB* strength of 2.53 MPa, a 12.65-fold increase (Fig. 2A). To clarify the role of activation time, we tracked changes in appearance and mechanical properties during pretreatment. As pretreatment progressed, the ignited biomass (Ignited-Biomass) gradually darkened from light brown to dark brown, while the additional color change became minimal beyond ~20 min (Fig. S3). Consistently, *IB* strength increased markedly with extended pretreatment time, corresponding to a gradual darkening of the BioFuse-Board’s color and a gradual decrease in thickness, while water absorption and thickness swelling rate decreased substantially. However, both properties peaked at 20 min and tended toward equilibrium (Fig. S4). When pretreatment time was extended to 30 min, both properties decreased slightly (Fig. 2B). Next, the hot-pressing stage was evaluated using Ignited-Biomass-20. To isolate the effect of pressing temperature, all samples in Fig. 2C were hot-pressed at a fixed pressure of 2 MPa for 30 min, while only the pressing temperature was varied. Under this identical pressing duration, increasing the temperature from 140 to 160 °C enhanced *IB* strength while reducing thickness swelling, whereas further increasing the temperature weakened the balance between cohesion and dimensional stability (Fig. 2C). Therefore, the *IB* values at different temperatures represent performance under the fixed 30-min condition, rather than independently optimized strengths at each temperature. The lower *IB* strength at 140 °C may result from insufficient interfacial fusion under this pressing duration, while the lower IB strength at 180 °C may reflect overtreatment under the same fixed-time condition. We then examined pressing time at 160 °C and 2 MPa. Extending the time from 20 to 30 min increased the *IB* strength to 2.53 MPa and maintained the 24-h thickness swelling at 10.05%, whereas further extension disrupted this balance (Fig. 2D). Similarly, increasing the pressure beyond 2 MPa under 160 °C and 30 min did not further improve the overall performance (Fig. 2E). Thus, 160 °C, 30 min, and 2 MPa were selected as a balanced processing condition within the investigated 1-factor processing window. Differential scanning calorimetry (DSC) and thermogravimetric analysis analyses further confirmed that the selected DES ignition and hot-pressing temperatures were within a thermally manageable processing range, with no substantial bulk mass loss observed at 120 or 160 °C (Fig. S5).

Within this processing window, the adhesive-free boards meet or exceed commercial standards for wood-based panels and show a marked advantage over most previously reported lignocellulosic bio-boards (Tables S1 and S2 and Fig. S6). This performance window is also manufacturable at larger scale, as demonstrated by the pilot-scale processing workflow and the corresponding BioFuse-Board panels produced under the same ignition–fusion protocol (Fig. S7). Thus, the DES ignition-triggered interfacial fusion strategy successfully turns loose lignocellulosic particles into structural-grade, adhesive-free boards.

Furthermore, the simultaneous improvement in *IB* strength and water resistance can be attributed to coupled densification and interfacial reconstruction. As pressing temperature, duration, and pressure increased, board density rose and internal voids were reduced (Fig. 2C and D and Fig. S8), which promoted load transfer across particle contacts and limited continuous pathways for water penetration (Figs. S9 and S10) [40]. Notably, the DES ignition-triggered interfacial fusion strategy mitigates the long-standing strength–water-resistance trade-off typical of binderless routes: At the condition delivering a 12.65-fold increase in *IB* strength, the 24-h thickness swelling decreases to 10.05% (Fig. 2A and G), whereas the directly hot-pressed control disintegrates after only 1 min of water immersion. Finally, the trends in Fig. S11A and B link these macroscopic outcomes to interfacial component redistribution during DES pretreatment. Composition analysis shows that DES treatment increases cellulose from 46.40% to 53.55%–54.18% while reducing hemicellulose from 20.17% to 10.31%–12.49%, with lignin decreasing only modestly (from 26.12% to 22.45%–23.74%). DES activation is consistent with redistribution of lignin- and hemicellulose-rich fractions toward particle surfaces, with part of the lignin becoming enriched at fiber peripheries. In parallel, redistributed hydrophobic lignin together with partial removal of hydrophilic hemicellulose synergistically improves water resistance. Time-resolved composition further supports this trend (Fig. S11B): Extending pretreatment from 10 to 30 min increases cellulose from 49.98% to 55.51%, while hemicellulose decreases from 14.87% to 10.31% and lignin declines from 24.28% to 22.74%. However, excessive dissolution at longer pretreatment times weakens particle integrity and increases the likelihood of collapse during pressing, consistent with the slight performance decline beyond 20 min.

To further decouple the contribution of migratable interfacial components from general densification during the ignition–fusion process, we performed a solvent-perturbation experiment using the optimally activated Ignited-Biomass before hot pressing (Fig. 2F and H). Compared to the optimal BioFuse-Board (water-washed, 2.53 MPa), the *IB* strength dropped to just 0.05 MPa after acetone washing (Fig. 2F). The corresponding water-immersion test showed the same trend: The acetone-washed board rapidly disintegrated within 1 min in water, similar to the directly hot-pressed untreated wood-powder control, whereas the water-washed BioFuse-Board maintained its integrity after immersion in water for 24 h (Fig. 2G and H). Because the ignition protocol already includes water washing to remove residual DES, the retained bonding strength and water stability after water washing indicate that the load-bearing interphase is not derived from residual DES. Instead, the collapse of both internal cohesion and water resistance after acetone washing indicates that acetone removes organosoluble, lignin-rich mobile components generated during DES ignition. These results provide perturbation-based evidence that migratable lignin-rich interfacial fractions are necessary precursors for subsequent interfacial network locking during hot pressing.

### DES-enabled interfacial ignition primes cell wall interfaces for fusion

**Fig. 3.**
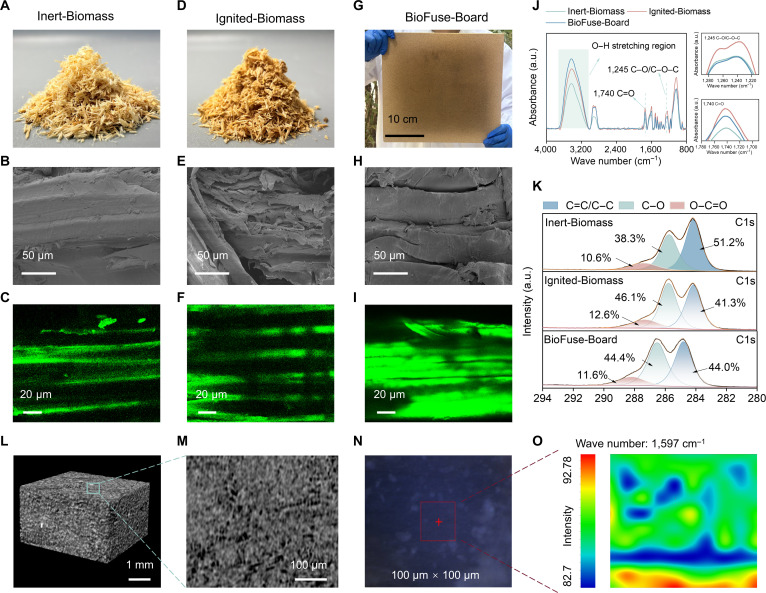
Deep eutectic solvent (DES) ignition-triggered interfacial fusion rewires interfacial chemistry and reconstruct lignocellulosic cell wall components. (A, D, and G) Photographs of Inert-Biomass, Ignited-Biomass and the resulting BioFuse-Board. (B, E, and H) Scanning electron microscopy images showing the evolution from smooth native surfaces to ignition-induced wall disruption/roughening, and then to fused, tightly contacted architectures after hot pressing. (C, F, and I) Lignin fluorescence (laser confocal scanning microscopy) across stages, showing redistribution during ignition and intensified, more continuous interfacial fluorescence after fusion. (J) Enlarged Fourier-transform infrared (FTIR) spectra of Inert-Biomass, Ignited-Biomass, and BioFuse-Board, with zoomed-in regions around 1,740 and 1,245 cm^−1^ to clarify the evolution of O–H stretching, carbonyl, and C–O/C–O–C-related environments during DES ignition and hot-pressing fusion. (K) High-resolution C1s x-ray photoelectron spectroscopy deconvolution of Inert-Biomass, Ignited-Biomass, and BioFuse-Board. Percentages indicate C1s-internal deconvolution proportions normalized within each C1s envelope. Survey-derived C/O atomic composition and C1s-internal deconvolution results are summarized in Table S4. (L and M) Three-dimensional microcomputed tomography reconstruction and representative slice showing densification and reduced void content in BioFuse-Board relative to the unfused control architecture. (N and O) FTIR imaging over a selected area and the corresponding chemical map at the lignin-associated band (1,597 cm^−1^), visualizing a spatially continuous lignin-rich interphase after fusion.

To elucidate the structural origin of DES ignition-triggered interfacial fusion-enabled performance, we tracked how the lignocellulosic cell wall and interparticle interfaces evolve during interfacial ignition and subsequent fusion using compositional analysis, scanning electron microscopy (SEM), and laser confocal scanning microscopy. Inert-biomass particles are yellowish brown, with dim lignin fluorescence that is largely uniform and continuous around fiber bundles, and they exhibit relatively flat, smooth surfaces (Fig. 3A to C). After DES activation, the substrate progressively darkens (Fig. 3D), consistent with a rapid redistribution of matrix polymers at the cell wall scale. Component analysis confirms this reorganization: lignin and hemicellulose contents decrease markedly, while cellulose increases in relative abundance, reflecting the different stabilities of the 3 components in DES (Fig. S11). SEM directly captures how ignition disrupts the native lignin and prepares the interface for fusion (Fig. 3E). Laser confocal scanning microscopy shows that the initially continuous lignin signal becomes brighter yet discontinuous after activation, indicating that part of the lignin migrates toward the fiber periphery and breaks the original uniform distribution (Fig. 3F). In parallel, SEM reveals a clear transition from smooth, intact fiber surfaces to roughened and more disordered walls (Fig. 3B, E, and H), consistent with partial deconstruction of the wall lamellae and the creation of an activated, more accessible interface. Importantly, these changes are not the final state. During the fusion stage, lignin fluorescence becomes strongly enhanced and more uniformly distributed, forming a compact and highly entangled appearance (Fig. 3I). SEM of the reconstructed fibers shows tight bonding and a densely cross-linked architecture in which previously fragmented elements appear fused into a coherent network (Fig. 3H). Fracture observations further support this consolidation, with continuous fiber–fiber contact and cohesive failure through a dense matrix rather than interfacial debonding (Figs. S8B and S12).

Bulk spectroscopy captures how DES ignition-triggered interfacial fusion alters interfacial chemistry from ignition to fusion. The enlarged Fourier-transform infrared (FTIR) spectra show changes in oxygen-containing functional groups during DES ignition and hot-pressing fusion (Fig. 3J). The broad O–H stretching band at 3,200 to 3,500 cm^−1^ reflects hydroxyl-rich environments and hydrogen bond interactions. The carbonyl band near 1,740 cm^−1^ and the C–O/C–O–C-related band near 1,245 cm^−1^ are observed in the activated and reconstructed samples, indicating the involvement of carbonyl-containing and oxygen-bridged environments in interfacial reconstruction. Because the 1,245 cm^−1^ region contains overlapping fingerprint vibrations, this band is interpreted together with x-ray photoelectron spectroscopy (XPS) rather than used alone as quantitative evidence for bond formation. XPS further resolves the chemical evolution at the outer cell wall region where interparticle contacts form (Fig. 3K). The survey-derived C1s contribution changes from 72.49% in Inert-Biomass to 65.59% in Ignited-Biomass and then to 72.17% in BioFuse-Board, with the remaining signal mainly arising from O1s (Table S4). After normalization within the C1s envelope, DES ignition increases oxygen-containing carbon environments, as C–C/C=C decreases from 51.2% to 41.3%, while C–O increases from 38.3% to 46.1% and O–C=O from 10.6% to 12.6%. This is accompanied by an increase in surface oxygen from 27.51% to 34.41%, indicating surface oxygen enrichment and chemical activation. During hot-pressing fusion, the surface oxygen content decreases to 27.83%, while the C1s-internal oxygenated carbon contributions remain prominent but slightly decrease, with C–O at 44.4% and O–C=O at 11.6%. This evolution suggests that hot pressing mainly consolidates and reorganizes the activation-generated oxygenated precursors into the bonding interphase, rather than introducing additional oxygen. Together with the FTIR signatures of carbonyl and C–O/C–O–C-related environments (Fig. 3J), the XPS results are consistent with interfacial locking through condensation/esterification and lignin-rich carbon-domain reconstruction.

Microstructural imaging then shows the architectural outcome of this chemistry. Three-dimensional microcomputed tomography reveals that BioFuse-Board formed via activation and reconstruction is dense and nearly void-free (Fig. 3L and M), whereas untreated boards retain a loose and porous architecture (Fig. S13). This reduction in porosity is consistent with the formation of continuous load-transfer pathways and suppressed transport channels for water ingress, and it complements the process-window trends in Fig. 2 where increasing consolidation improves both cohesion and water resistance. Finally, FTIR imaging connects the densified architecture to the distribution of the lignin-rich bonding phase. Imaging at the lignin-associated band near 1,597 cm^−1^ shows continuous and spatially uniform lignin-rich signals across the BioFuse-Board surface (Fig. 3N and O), supporting a percolating lignin-enriched interphase that bridges adjacent fibers rather than isolated lignin patches. Taken together, the chemical evolution (Fig. 3J and K), the densified internal architecture (Fig. 3L and M), and the spatial continuity of lignin-rich regions (Fig. 3N and O) all point to a reconstructed network in which lignin-rich components are redistributed toward particle contact regions and then consolidated during hot pressing into a continuous interfacial bonding network that bridges adjacent particles/fibers and enables load transfer.

**Fig. 4.**
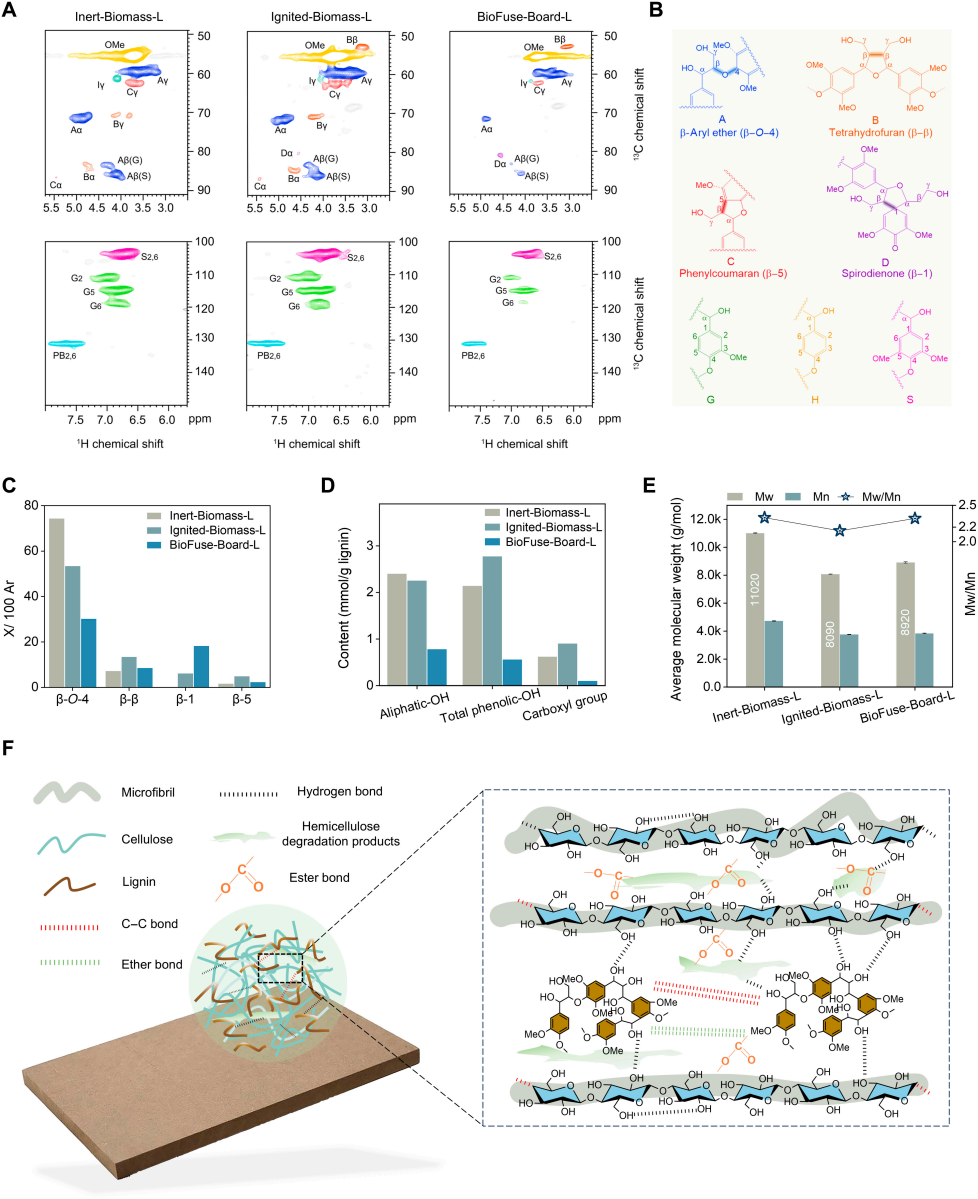
Two-stage lignin reprogramming underlies ignition-triggered interfacial fusion. (A) Two-dimensional heteronuclear single quantum coherence (2D-HSQC) nuclear magnetic resonance (NMR) spectra of lignin isolated from Inert-Biomass, Ignited-Biomass, and BioFuse-Board, showing stage-dependent changes in interunit linkages. (B) Representative possible substructures of lignin for explaining changes in 2D-HSQC NMR spectra. (C) Quantification of major interunit linkages (including β–*O*–4 and condensed linkages), evidencing depolymerization during ignition and increased condensation signatures after fusion. (D) Reactive-site quantification (aliphatic-OH, total phenolic-OH, and carboxyl groups), showing reactive-site enrichment during ignition and depletion upon network consolidation during fusion. (E) Molecular weight evolution (Mw, Mn, and dispersity), indicating formation of a more mobile lignin intermediate after ignition and partial recovery consistent with interfacial network formation after fusion. (F) Proposed adhesive-free bonding mechanism during deep eutectic solvent (DES) ignition-triggered interfacial fusion. DES ignition partially depolymerizes hemicellulose and lignin, cleaves β–*O*–4 linkages, and generates mobile lignin-/hemicellulose-derived fragments containing reactive hydroxyl and carboxyl groups. During hot pressing, these fragments are consolidated with the cellulose scaffold into a reconstructed interfacial network through possible ester-like O–C=O linkages, C–O/C–O–C-related interactions, lignin-rich C–C condensation, hydrogen bonding, and physical interlocking.

Viewed together, DES ignition-triggered interfacial fusion achieves this interfacial transformation without disrupting the crystalline cellulose scaffold, as x-ray diffraction shows that all samples retain the characteristic cellulose I reflections (Fig. S14). The crystallinity index increases from 49.10% (Inert-Biomass) to 53.44% after ignition (Ignited-Biomass) and then slightly decreases to 51.58% in BioFuse-Board, consistent with ignition reducing amorphous contributions and fusion consolidating an interphase while preserving the crystalline cellulose scaffold. These observations motivate the molecular-level analysis in Fig. 4, where we quantify how lignin linkages and functional groups evolve across the same 2 stages, consistent with lignin reprogramming from a mobilized intermediate during activation to a locked bonding network during reconstruction.

### Lignin redistribution and interfacial network locking build a load-bearing bonding interphase

To connect the molecular changes in lignin to the structural-grade performance of BioFuse-Board, we examined how DES ignition-triggered interfacial fusion converts native lignin into an interfacial bonding phase that first redistributes during activation and is then locked into an interface network during hot pressing. Using methods described in previous literature [41,42], structurally intact lignin (double enzymatic lignin [DEL]) was isolated from inert biomass, postcombustion biomass, and reconstituted BioFuse panels. It was characterized using 2-dimensional heteronuclear single quantum coherence nuclear magnetic resonance (2D-HSQC NMR) spectroscopy, combined with semiquantitative bond-linking structural analysis and molecular weight determination (Fig. 4A to E). Across these datasets, the DES ignition-triggered interfacial fusion strategy follows a 2-stage trajectory in which activation generates a mobile, reactive intermediate and reconstruction consumes reactive sites while consolidating interfacial connectivity. In the 2D-HSQC spectra, correlations assigned to β–*O*–4 motifs weaken after activation, including the Aβ(G) and Aβ(S) signals (δ_C_/δH = 83.4/4.38 and 85.8/4.12) and the Aα cross peak at δC/δH = 71.8/4.86 (Fig. 4A). Quantitative linkage analysis shows the same trend. The β–*O*–4 content drops from 74.50/100 Ar in Inert-Biomass-L to 53.57/100 Ar in Ignited-Biomass-L (Fig. 4C). These scission events are accompanied by a pronounced shift in reactive-site balance. Total phenolic-OH increases from 2.1 to 2.8 mmol g^−1^ lignin, and carboxyl groups increase from 0.6 to 0.9 mmol g^−1^ lignin, while aliphatic-OH decreases slightly from 2.4 to 2.2 mmol g^−1^ lignin (Fig. 4D). At the molecular scale, the activated lignin becomes smaller and more mobile. Mw decreases from 11,020 to 8,090 g mol^−1^, with Mn decreasing from 4,740 to 3,770 g mol^−1^, while dispersity remains around 2.15 (Fig. 4E). Aromatic-unit analysis shows only a minor increase in S/G during activation, from 1.26 to 1.31, with the relative abundance of S and G units changing from 55.77/100 Ar and 44.23/100 Ar in Inert-Biomass-L to 56.65/100 Ar and 43.35/100 Ar in Ignited-Biomass-L (Fig. S15). This modest shift suggests limited change in overall aromatic-unit composition. More direct evidence for activation-induced lignin reprogramming comes from the pronounced β–*O*–4 cleavage, reduced molecular weight, and increased phenolic and carboxyl contents, which together indicate formation of a more depolymerized and chemically accessible intermediate consistent with the redistribution observed in the preceding sections.

Reconstruction locks redistributed lignin into an interfacial network. After hot pressing, lignin chemistry shifts from mobility generation to network consolidation. Linkage analysis shows that condensed structures become more prominent in BioFuse-Board-L, most notably a strong rise in β–1 content to 18.36/100 Ar compared with 6.27/100 Ar in the activated lignin, alongside increases in β–β and β–5 linkages (Fig. 4C). Molecular weights partially recover, with Mw increasing to 8,920 g mol^−1^ and Mn rising to 3,850 g mol^−1^ relative to the activated intermediate (Fig. 4E), consistent with condensation and partial repolymerization during reconstruction. In parallel, the reactive-site pool created during activation is substantially depleted in the final board. Total phenolic-OH drops to 0.55 mmol g^−1^ lignin and carboxyl groups decrease to 0.1 mmol g^−1^ lignin, indicating that the newly generated sites are not merely accumulated at interfaces but are consumed as interfacial connectivity develops (Fig. 4D). The S/G ratio further increases to 2.23 in BioFuse-Board-L, with the relative abundance of S units rising to 69.00/100 Ar and that of G units decreasing to 31.00/100 Ar (Fig. S15), indicating a substantial change in aromatic-unit distribution during reconstruction. More direct evidence for interfacial consolidation comes from the increase in condensed linkages, the partial recovery of molecular weight, and the marked depletion of phenolic and carboxyl groups after hot pressing.

To further investigate the dynamic processes underlying interfacial chemical evolution, we performed 2D-correlation FTIR analysis using the state-resolved series of Inert-Biomass, Ignited-Biomass, and BioFuse-Board (Fig. S16). The synchronous map shows strong auto-peaks and cross-peaks in the O–H stretching region, carbonyl/ester region, aromatic lignin region, and C–O/C–O–C region, indicating that hydrogen bond reorganization, carbonyl evolution, lignin enrichment, and polysaccharide-associated skeletal changes are strongly coupled during ignition and fusion. The asynchronous map further reveals that these spectral responses are not fully simultaneous. Hydroxyl- and carbonyl-related bands respond earlier, followed by aromatic lignin vibrations and then C–O/C–O–C skeletal rearrangements. This dynamic process follows a 2-stage mechanism: The DES first weakens the hydrogen bonding regions of the unstable hemicellulose domains, forming a precursor state suitable for interfacial bonding, whereas subsequent hot-pressing treatment promotes the polycondensation of the lignin-rich phase and solidifies and locks the interfacial network. Thus, 2D-correlation FTIR provides dynamic spectroscopic support for the proposed ignition–fusion mechanism, complementing the static molecular evidence from 2D-HSQC NMR, reactive-site quantification, and molecular weight analysis.

The mechanism summarized in Fig. 4F captures how DES ignition and hot-pressing fusion translate molecular activation into interfacial locking. During DES ignition, the acidic ChCl/1,4-butanediol/AlCl_3_ system partially depolymerizes the lignocellulosic matrix, including cleavage of lignin β–*O*–4 linkages and partial degradation of hemicellulose. This process lowers lignin molecular weight and increases phenolic hydroxyl and carboxyl groups, thereby generating mobile lignin-/hemicellulose-derived fragments with enhanced interfacial reactivity. These activated fragments can redistribute toward particle contact regions. During subsequent hot pressing, the redistributed components are consolidated into a load-bearing bonding interphase. Carboxyl groups generated during ignition may react with hydroxyl groups from cellulose and hemicellulose to form ester-like O–C=O linkages, while C–O/C–O–C-related interactions, lignin–lignin condensation/C–C connectivity, strengthened hydrogen bonding, and physical interlocking also contribute to interfacial network locking. The marked depletion of phenolic hydroxyl and carboxyl groups after hot pressing, together with the increase in condensed lignin linkages and partial recovery of molecular weight, supports the conversion of the activation-generated precursor pool into a locked interfacial network. Therefore, the adhesive-free bonding of BioFuse-Board is attributed to a cooperative mechanism involving DES-induced activation/redistribution followed by hot pressing-induced chemical and physical interfacial consolidation, rather than to residual DES or a single bonding mode alone. Compared with previously reported adhesive-free or bio-based board strategies based mainly on thermal self-bonding, steam explosion, oxidation, acid/alkali activation, or externally added bio-based adhesives, the present ignition–fusion strategy programs the interface using native cell wall components. DES ignition generates a mobile lignin/hemicellulose-derived intermediate at particle surfaces, while hot pressing locks this intermediate into a connected bonding interphase. Therefore, the bonding mechanism differs from simple densification or externally supplied adhesive bonding: It relies on in situ redistribution and reconstruction of native components into a load-bearing interfacial network (Table S1).

### Ignition–fusion as a closed-loop platform: Reagent reuse, stream valorization, and board remanufacturing at system scale

**Fig. 5.**
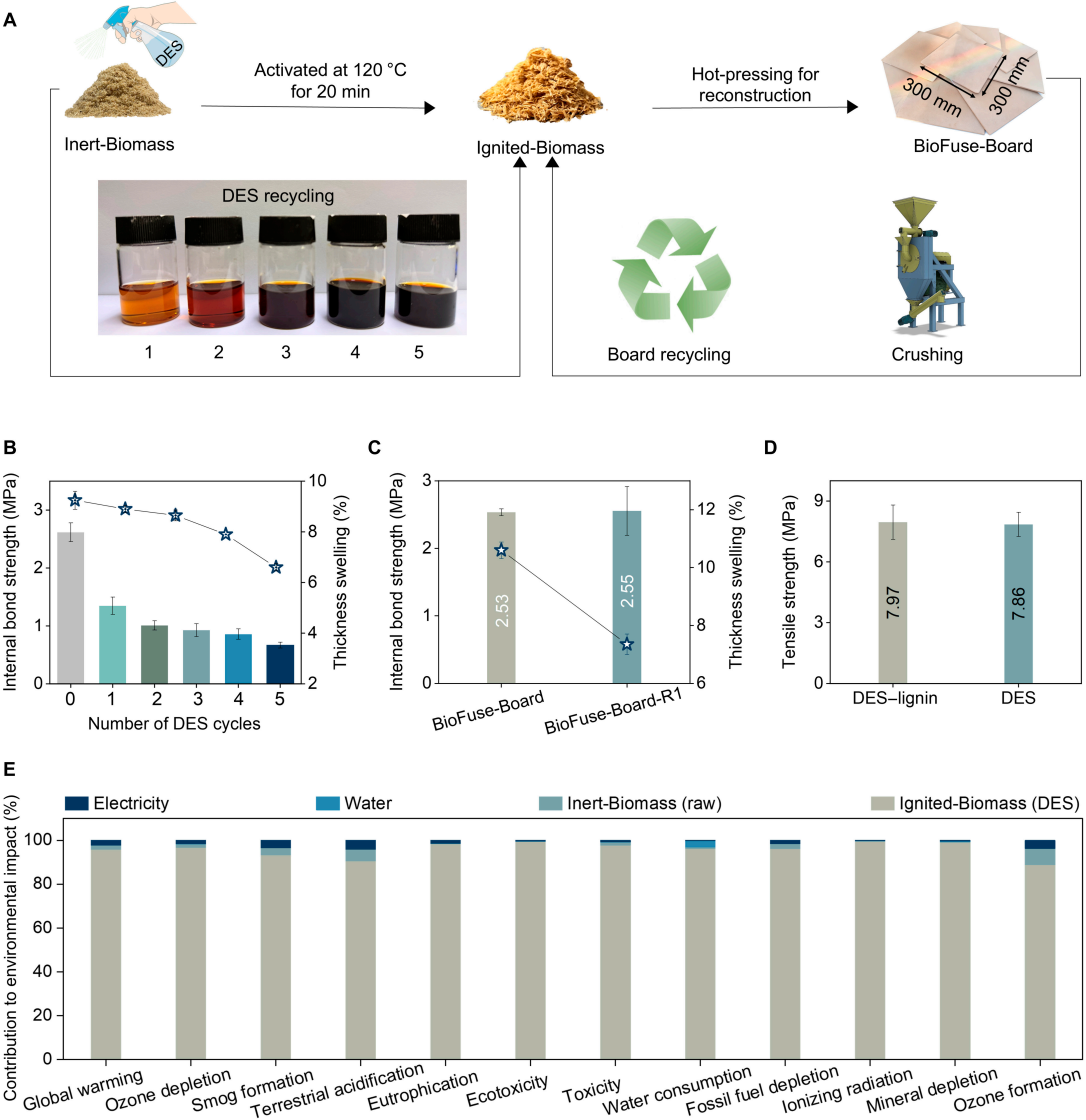
Multilevel circularity and system-level performance enabled by ignition-triggered interfacial fusion. (A) Closed-loop concept integrating deep eutectic solvent (DES) reuse and board recycling alongside the ignition-to-fusion manufacturing route. (B) Recyclability of the reagent loop: internal bond (*IB*) strength (bars) and thickness swelling (line) across repeated DES reuse cycles. (C) Product-level remanufacturing: comparison of *IB* strength (bars) and thickness swelling (line) between the original BioFuse-Board and the reprocessed BioFuse-Board-R1. (D) Valorization of process streams into DES-derived adhesive formulations, evaluated by tensile strength. (E) Cradle-to-gate impact contribution analysis highlighting the relative contributions of electricity, water, and material streams to multiple environmental categories. Error bars, SD.

DES ignition-triggered interfacial fusion is designed to close loops at the reagent, product, and process-stream levels, so circularity is embedded in the manufacturing architecture rather than appended at end of life. The overall circular workflow is illustrated in Fig. 5A and is further detailed by the remanufacturing loop (Fig. S17) and the closed-loop process schematic that couples DES and water recycling with stream valorization (Fig. S18). At the reagent-loop level, recovered DES remains functional across multiple reuse cycles. Reusing the DES progressively reduces *IB* strength from 1.35 MPa in the first run to 0.70 MPa by the fifth recycle, while thickness swelling remains controlled and decreases from 9.0% to 6.0%–7.0% over the same sequence (Fig. 5B). This paired trend indicates that the process can accommodate repeated reagent circulation while retaining dimensional stability, although bond strength progressively declines with reuse. The results therefore define a bounded operating window for circular reagent management rather than unlimited reuse. The process schematic in Fig. S18 clarifies how this is achieved in practice. DES ignition-triggered interfacial fusion couples interfacial activation with lignin regeneration, followed by separation that enables DES recovery and recycling, while the water stream is also routed for reuse, reducing net discharge and enabling a closed-loop liquid management strategy.

At the product-loop level, DES ignition-triggered interfacial fusion strategy enables remanufacturing by treating interfacial connectivity as regenerable. After service, BioFuse-Board can be crushed and re-pressed into a regenerated product (BioFuse-Board-R1), as visualized in the remanufacturing schematic (Fig. S17) and the board-recycling pathway in Fig. 5A. Importantly, remanufacturing preserves structural-grade cohesion. *IB* strength remains essentially unchanged, increasing from 2.53 MPa for the original board to 2.55 MPa for BioFuse-Board-R1, while thickness swelling decreases from 10.05% to 7.00% (Fig. 5C). This behavior contrasts with conventional thermoset-bonded boards, where irreversible networks typically force recycling into downcycling. In this strategy, the bonding phase is built primarily from redistributed native components and locked during pressing, which appears to preserve reprocessability and helps explain the retained interfacial cohesion after remanufacturing. DES ignition-triggered interfacial fusion strategy also closes a process-stream loop by converting spent streams into usable products rather than waste. The process schematic (Fig. S18) explicitly identifies a valorization pathway in which a DES-related stream is directed toward a DES-adhesive formulation, aligning with the measured adhesive performance. The resulting formulations achieve tensile strengths of 7.97 MPa (DES–lignin) and 7.86 MPa (DES) (Fig. 5D), demonstrating that streams generated within the loop can retain functional value and broaden the circularity portfolio beyond the board itself.

Finally, technoeconomic and environmental analyses converge on the same design lever: operating the reagent loop efficiently. TEA (Table S3) estimates a total material-and-energy cost of ¥1,773.2 /t^−1^ board (US$249.6 t^−1^) for producing 1 ton of BioFuse-Boards under the optimized Ignited-Biomass-20 condition, in which the dominant contribution comes from the recyclable DES inventory (¥822.48 t^−1^ board), while the biomass feedstock contributes ¥600 t^−1^, electricity contributes ¥348.05 t^−1^, and industrial water is negligible (¥2.7 t^−1^). Consistently, cradle-to-gate LCA shows that the activated-biomass stage associated with DES use dominates most impact categories, whereas electricity and water contribute comparatively small fractions (Fig. 5E), indicating that further footprint reduction depends less on pressing energy and more on extending DES lifetime, minimizing make-up losses, and improving the sourcing and synthesis profile of the recyclable chemistry. To place these system-level results in context, BioFuse-Board was further compared with conventional resin-bonded wood-based panels and representative adhesive-free/bio-based lignocellulosic board systems (Table S5). Conventional panels benefit from mature production infrastructure and low-cost adhesive systems, but they commonly rely on fossil-derived thermoset binders, some of which are associated with formaldehyde-related concerns. BioFuse-Board avoids externally added fossil-derived adhesives and integrates DES recovery, stream valorization, and product-level remanufacturing. However, the TEA and LCA results indicate that the DES-related activation stage remains the dominant cost and environmental contributor, suggesting that future improvement should focus on extending DES lifetime, reducing make-up losses, improving solvent recovery, optimizing solvent sourcing, and lowering process energy demand. Together, these results position DES ignition-triggered interfacial fusion as a manufacturable circular strategy whose economic and environmental performance is governed primarily by how effectively the closed-loop reagent architecture is operated, in line with the multicycle reuse demonstrated in Fig. 5B. Collectively, Fig. 5 shows that DES ignition-triggered interfacial fusion strategy embeds circularity into the process architecture. Reagent recycling remains viable, remanufacturing preserves structural performance, and waste streams retain functional value, so recyclability becomes an intrinsic manufacturing attribute rather than a compromise imposed after the fact.

## Conclusion

In brief, this work addresses a central challenge in lignocellulosic structural boards: how to form strong, water-resistant, yet remanufacturable interfacial bonding from native components without relying on external thermoset adhesives. We show that a minimal amount of recyclable DES can program loose poplar particles through an ignition–fusion sequence, transforming them into adhesive-free BioFuse-Boards with structural-grade performance, achieving an *IB* strength of 2.53 MPa and a 24-h thickness swelling of 10.05%. Mechanistically, ignition creates an interface-ready state by promoting β–*O*–4 cleavage, lowering lignin molecular weight, enriching reactive sites, and mobilizing native lignin and hemicellulose toward particle contact zones, whereas subsequent fusion reconstructs these redistributed components into a continuous bonding interphase through condensation, possible ester-like O–C=O formation, lignin-rich C–C connectivity, and strengthened hydrogen bonding with cellulose. Beyond performance, the same ignition–fusion architecture embeds circularity into manufacturing through DES recovery and reuse, valorization of process streams into wood adhesives, and end-of-life remanufacturing into regenerated products that retain structural-grade cohesion. Together, these findings establish DES ignition-triggered interfacial fusion as a practical and generalizable strategy for circular lignocellulosic structural materials manufacturing, opening a new path to structural-grade, adhesive-free, and remanufacturable boards by programming regenerable interfacial bonding from native components.

## Materials and Methods

### Chemicals

All commercial chemicals were analytical reagents and were used without further purification. 1,4-Butanediol (98%) and anhydrous aluminum chloride (AlCl_3_) were obtained from Macklin Chemical Co. Choline chloride (ChCl, 98%) was purchased from Merck Chemical Co. Polyvinyl alcohol (Mw = 1,750 ± 50) was supplied by Shanghai Aichun Biological Technology Co., Ltd., China. Poplar wood powder was obtained from Tianlaizhilin Wood Industry (Linyi, Shandong Province, China). Lignin was provided by Shandong Longli Biotechnology Co., Ltd., China.

### Preparation of Ignited-Biomass

The DES was prepared by mixing choline chloride, 1,4-butanediol, and anhydrous aluminum chloride in a molar ratio of 1:2:0.04. The mixture was stirred and heated at 80 °C until a homogeneous, transparent liquid formed. Subsequently, 30 g of DES (corresponding to 1.5 times the mass of the poplar wood powder) was mixed with 9 g of water (30% of the DES mass) to form an aqueous DES solution, ensuring uniform distribution within the lignocellulosic matrix. This solution was evenly sprayed onto 20 g of lignocellulosic particles placed in a tray. After thorough mixing, the treated particles were heated in an oven at 120 °C for varying durations (10, 20, and 30 min). Following this ignition stage, distilled water was added to the particles at a 1:5 (v/v) ratio, and the mixture was stirred at 500 rpm for 2 h to facilitate DES recovery. The solids were then filtered, washed with distilled water to remove residual DES, and dried at 63 °C. The resulting materials were designated as Ignited-Biomass-10, Ignited-Biomass-20, and Ignited-Biomass-30, according to their respective heating times.

### Preparation of BioFuse-Board

The Ignited-Biomass-20 (hereinafter referred to as Ignited-Biomass) was left in air for a period to equilibrate its moisture content (adjusting the wood powder moisture content to approximately 3%). Subsequently, 20 g of Ignited-Biomass was poured into a 4 cm × 8 cm stainless steel mold to obtain the desired shape. Finally, the material was hot-pressed at 160 °C under a pressure of 2 MPa for 30 min, to produce the BioFuse-Board. Some of the BioFuse-Board was cut into small pieces with dimensions of 5 × 5 × 5 mm^3^ using a small table saw for the following characterizations.

### Mechanical strength tests of BioFuse-Board

The *IB* strength was tested according to the Chinese National Standard GB/T 17657-2022. Specimens with dimensions of 40 mm × 40 mm × (3 to 5 mm) were prepared for the test. The length and width of all specimens were (40 ± 1) mm. A hot-melt adhesive was evenly applied to the surface of the loading heads, which were then bonded to both surfaces of each specimen. The bonded assembly was placed in the clamping device of a universal testing machine. A constant loading rate was applied until specimen failure, and the maximum load was recorded with an accuracy of ±1 % of the measured value. Five replicates were tested for each sample group. The *IB* strength (*α*_⊥_) was calculated using Eq. (1) and reported with an accuracy of 0.01 MPa.

$$\alpha_{⊥}=F_{\max}/l\times b$$ (1)

where *α*_⊥_ is the *IB* strength of the specimen, in megapascals (MPa); *F*_max_ is the maximum load at which the specimen breaks, in newtons (N); *l* is the length of the specimen, in millimeters (mm); and *b* is the width of the specimen, in millimeters (mm).

### Water resistance (thickness swelling ratio) tests

The thickness swelling of the specimen was determined in accordance with the Chinese National Standard GB/T 17657-2022. The diagonal intersection point on each specimen was marked and used as the thickness measurement point, and the initial thickness (*t*_1_) was recorded. After immersion in water at room temperature for 24 h, the thickness at the same central point (*t*_2_) was measured again. The thickness swelling ratio was then calculated using Eq. (2).

$$T=\left(t_{2}-t_{1}\right)/t_{1}\times 100\%$$ (2)

where *T* is the water-absorption thickness swelling ratio (%); *t*_2_ is the thickness of the specimen after immersion in water, in millimeters (mm); and *t*_1_ is the thickness of the specimen before immersion in water, in millimeters (mm).

### FTIR spectroscopy

FTIR spectra of the samples were recorded in transmission mode using a Thermo Nicolet iN10 FTIR spectrometer. The spectra were collected over the wavenumber range of 4,000 to 700 cm^−1^.

### X-ray diffraction

Wide-angle x-ray diffraction patterns of the samples were obtained using an x-ray diffractometer (Bruker D8, *λ* = 0.154 nm). The instrument was operated at 40 kV and 40 mA. The diffraction patterns were recorded over a 2θ range of 5° to 40° at a scanning rate of 2° min^−1^.

### Scanning electron microscopy

The surface and fracture-surface microstructures of Inert-Biomass, Ignited-Biomass, and BioFuse-Board were observed using SEM (Regulus 8100, Hitachi). The observations were performed under high-vacuum mode at an accelerating voltage of 10.0 kV. Before SEM observation, the dried samples were sputter-coated with a conductive metal layer.

### Laser scanning confocal microscopy

The lignin distribution in the samples was visualized using laser scanning confocal microscopy (TCS SP8, Leica). The samples were excited at 488 nm, and the emission signal was collected over the range of 500 to 600 nm.

### FTIR microscopy and chemical imaging

FTIR microscopy was performed using a Nicolet iN10 FTIR microscope (Thermo Fisher Scientific). Spectra were recorded over the range of 4,000 to 800 cm^−1^ with a spectral resolution of 4 cm^−1^. FTIR microscopic imaging was conducted over an area of 50 × 50 μm^2^, and the chemical map at 1,597 cm^−1^ was used to visualize the lignin-associated distribution in the sample [43].

### Differential scanning calorimetry

Thermal transitions of the lignocellulosic particles were analyzed using DSC (DSC 60 Plus, Shimadzu). Before DSC measurement, the samples were dried in an oven at 65 °C to remove excess water. The dried samples were sealed in aluminum crucibles with lids and heated from room temperature to 200 °C at a heating rate of 10 °C min^−1^ under a nitrogen atmosphere with a flow rate of 10 ml min^−1^, followed by a 5-min isothermal hold at 200 °C.

### Thermogravimetric analysis

The thermal stability of the lignocellulosic particles was evaluated using thermogravimetric analysis. The samples were heated from 30 to 750 °C at a heating rate of 10 °C min^−1^ under a nitrogen atmosphere with a flow rate of 50 ml min^−1^.

### X-ray photoelectron spectroscopy

XPS (K-Alpha+, Thermo Scientific) was performed using a monochromatic Al Kα x-ray source (1,486.6 eV, 100 W). Inert-Biomass, Ignited-Biomass, and BioFuse-Board were ground into 40- to 60-mesh powders before analysis. XPS was used to analyze the surface elemental composition and chemical environments of the samples.

### Chemical composition analysis

The bulk chemical compositions of Inert-Biomass, Ignited-Biomass, and BioFuse-Board were determined according to the National Renewable Energy Laboratory sulfuric acid hydrolysis protocol [44]. Monomeric sugars were quantified by high-performance anion-exchange chromatography. Acid-soluble lignin was determined by ultraviolet–visible spectroscopy, and acid-insoluble lignin was determined gravimetrically from the solid residue.

### Contact angle tests

The contact angle of the BioFuse-Board was analyzed using an optical contact angle goniometer (model). A camera recorded the contact angle and shape of the water droplet at 0, 5, 10, and 20 s to evaluate the surface hydrophobicity of the samples.

### Preparation of DEL from Inert-Biomass, Ignited-Biomass, and BioFuse-Board

DEL samples were isolated from Inert-Biomass, Ignited-Biomass, and BioFuse-Board using the same ball-milling/double-enzymatic-hydrolysis protocol as reported in the previous study [45].

### NMR spectral analysis of DEL

NMR spectra of the isolated DEL samples were acquired on a Bruker AVIII 400 MHz spectrometer at 25 °C in DMSO-*d*6. Quantitative 2D-HSQC experiments were performed following established procedures [46,47]. Quantitative ^31^P NMR spectra were obtained following literature methods [48,49].

### Life cycle assessment

This study aimed to assess the environmental impacts of producing 1 t of board through an LCA analysis using SimaPro 9.0, in accordance with ISO 14040/14044.

## Data Availability

The data that support the findings of this study are available from the corresponding authors upon reasonable request.
