# Supplementary material for: Deep Eutectic Solvents Ignition-Triggered Interfacial Fusion for Structural-Grade Lignocellulosic Boards
Source: Research (Wash D C). 2026 Jul 17;9:1365. doi: 10.34133/research.1365 (PMC13376383; doi:10.34133/research.1365)
Supplement: Supplementary 1 — Supplementary Methods Figs. S1 to S18 Tables S1 to S5 [file research.1365.f1.docx]

**Supplementary Materials for**

***Deep Eutectic Solvents-Ignition-Triggered Interfacial Fusion for Structural-Grade Lignocellulosic Boards***

*Huiru Yue^1‡^, Cheng Zuo^1‡^, Xinyi Hui^1^, Jia-Long Wen^1,2^*, Tong-Qi Yuan^1,2^**

*^1^State Key Laboratory of Efficient Production of Forest Resources, Beijing Key Laboratory of Lignocellulosic Chemistry, Beijing Forestry University, Beijing 100083, China.*

*^2^Hebei Key Laboratory of Agricultural and Forestry Biomass Materials Science and Application, Beijing Forestry University, Xiong'an 070001, China.*

*^‡^These authors contributed equally to this work.*

**Corresponding authors:* [*wenjialong@bjfu.edu.cn*](mailto:wenjialong@bjfu.edu.cn) *(J.W.);* [*ytq581234@bjfu.edu.cn*](mailto:ytq581234@bjfu.edu.cn) *(T.Y.)*

**Table of Contents**

**Supplementary Methods**

Preparation of adhesive and strength testing

Density determination of the BioFuse-Board

Preparation of double enzymatic lignin (DEL)

Acetylation of DEL and molecular-weight determination

Nuclear magnetic resonance (NMR) spectral analysis of DEL

**Supplementary Figures S1 to S18**

**Fig. S1.** Laboratory-scale preparation of adhesive-free BioFuse-Board.

**Fig. S2.** Photographs of molds.

**Fig. S3.** Visual evolution of furnish appearance during DES ignition at different times, consistent with progressive interfacial reprogramming.

**Fig. S4.** BioFuse-Board prepared at different DES activation time.

**Fig. S5.** Thermal analysis of Inert-Biomass, Ignited-Biomass, and BioFuse-Board.

**Fig. S6.** Comprehensive performance of our BioFuse-Board compared with commercial wood-based panels and reported lignocellulosic composites.

**Fig. S7.** Pilot-scale preparation of BioFuse-Board.

**Fig. S8.** Control of BioFuse-board density and its link to performance.

**Fig. S9.** Evolution of cell-wall morphology with different DES activation time during ignition and fusion.

**Fig. S10.** Contact angles of pure water after 25 seconds on the surface of BioFuse-Board.

**Fig. S11.** Changs in cellulose, hemicellulose and lignin contents during different activation time and fusion.

**Fig. S12.** Fracture surface morphology of BioFuse-Board after internal bond strength testing.

**Fig. S13.** Three-dimensional micro-CT reconstruction of BioFuse-Board and non-DES-activated control samples.

**Fig. S14.** The XRD spectrogram of Inert-Biomass, Ignited-Biomass and the BioFuse-Board.

**Fig. S15.** Changes in S- and G-type lignin units during ignition and fusion.

**Fig. S16.** Two-dimensional correlation FTIR analysis during DES ignition and fusion.

**Fig. S17.** Closed-loop recycling of BioFuse-Board.

**Fig. S18.** DES ignited Inert-Biomass interfacial process for preparing Ignited-Biomass and DES recycling flow chart.

**Supplementary Tables S1 to S5**

**Table S1**. Comparison of performance, processing strategy, and bonding mechanism of BioFuse-Board with representative adhesive-free and bio-based lignocellulosic board systems.

**Table S2.** Requirements for internal bond strength and 24-h thickness swelling of commercial wood-based panels in different standards.

**Table S3**. Estimated raw-material cost for producing 1 ton of BioFuse-Board with Ignited-Biomass−20.

**Table S4**. XPS survey atomic composition and C1s-internal deconvolution results of Inert-

Biomass, Ignited-Biomass, and BioFuse-Board.

**Table S5**. Comparison of techno-economic and environmental features of BioFuse-Board with conventional wood-based panels and related adhesive-free/bio-based board systems.

**Supplementary Methods**

**Preparation of Adhesive and strength testing**

A 10 wt% aqueous polyvinyl alcohol (PVA) solution was prepared by dissolving 10 g of PVA in 90 g of water under heating and stirring at 90 ^o^C until a clear, bubble-free solution was obtained. The recovered DES from the fifth cycle was then blended with the PVA solution, as well as with a mixture of PVA solution and lignin, at mass ratios of 1:1 and 1:1:0.1 (DES: PVA: lignin), respectively, to prepare composite wood adhesives. Each blend was stirred at 450 rpm and 90 ^o^C to ensure homogeneity. For lap shear testing, beechwood substrates (20 mm × 80 mm × 5 mm) were used. Approximately 60 mg of adhesive was applied to an overlap area of 20 mm × 10 mm. Six specimens were assembled and hot‑pressed together under a pressure of 1.1 MPa at 160 ^o^C for 20 min. All tests were conducted in duplicate to ensure reproducibility. Lap shear strength was measured using a universal testing machine (UTM 6530, Shenzhen Suns Technology Co., Ltd., China). The adhesive strength was calculated by dividing the maximum load (N) by the bonded overlap area (mm^2^).

**Density determination of the BioFuse-Board**

The length and width of all specimens used for density calculation were (40 ± 1) mm. The mass of the Biofuse-Board specimens was weighted with an accurately of 0.01 g. The length and width of the specimens were measured at the midpoint of each side, with an accuracy of 0.1 mm. The density (*ρ*) of the specimens was calculated using Equation (1) with an accuracy of 0.01 g/cm^3^:

$\text{ρ=}\text{m}/\left( \text{l ×b ×t} \right)\text{×}\text{ }\text{1000}$ (1)

In the formula:

*ρ* — Specimen density, unit: gram per cubic centimeter (g/cm^3^);

*m* — Specimen mass, unit: gram (g)

*l* — Specimen length, unit: millimeter (mm)

*b* — Specimen width, unit: millimeter (mm)

*t* — Specimen thickness, unit: millimeter (mm)

**Preparation of double enzymatic lignin (DEL) from Inert-Biomass, Ignited-Biomass, and BioFuse-Board**

5 g of each dried and ball-milled sample was suspended in 100 mL of acetate buffer (pH = 4.8), and 2.5 mL of cellulase (100 FPU mL⁻¹) was added. The mixture was then incubated in a rotary shaker at 50 ^o^C and 150 rpm for 48 h. Subsequently, the mixture was centrifuged, and the residual solids were thoroughly washed with acetate buffer (pH = 4.8) and hot water to remove hydrolyzed carbohydrates, followed by freeze-drying. Finally, the dried residual solids were ball-milled again for 2 h and subjected to enzymatic hydrolysis once more following the procedure described above. After washing with warm water and freeze-drying, the double ball-milled enzymatic lignin (DEL) sample was obtained.

**Acetylation of DEL and molecular-weight determination**

To improve DEL solubility in tetrahydrofuran (THF) for gel permeation chromatography (GPC), lignin samples were acetylated prior to analysis. Approximately 30 mg of oven-dry DEL was dissolved in 3 mL of a dimethyl sulfoxide/1-methylimidazole mixture (2:1 v/v) and stirred at room temperature in the dark for 12 h. Acetic anhydride (1.0 mL) was then added and the reaction was continued for 2 h. The mixture was slowly poured into 100 mL of acidic water (pH 2.0) to precipitate the acetylated lignin, which was collected by centrifugation. The number-average (M_n_) and weight-average (M_w_) molecular weights of the acetylated DEL were determined by GPC (Agilent 1200, Agilent Technologies, USA) equipped with a UV detector set at 240 nm.

**Nuclear magnetic resonance** (**NMR) spectral analysis of DEL**

20 mg of lignin was dissolved in 500 µL of anhydrous pyridine/deuterated chloroform (1.6:1 v/v), followed by addition of 100 µL cyclohexanol solution (10.85 mg mL^-1^ in the same solvent mixture) as the internal standard and 100 µL chromium (III) acetylacetonate solution (5 mg mL^-1^) as the relaxation agent. The mixture was then reacted with 100 µL of the phosphitylating reagent 2-chloro-4,4,5,5-tetramethyl-1,3,2-dioxaphospholane (TMDP) for 10 min and transferred to a 5 mm NMR tube for ^31^P NMR analysis. All NMR measurements were performed in duplicate.

**Supplementary Figures S1 to S18**

**
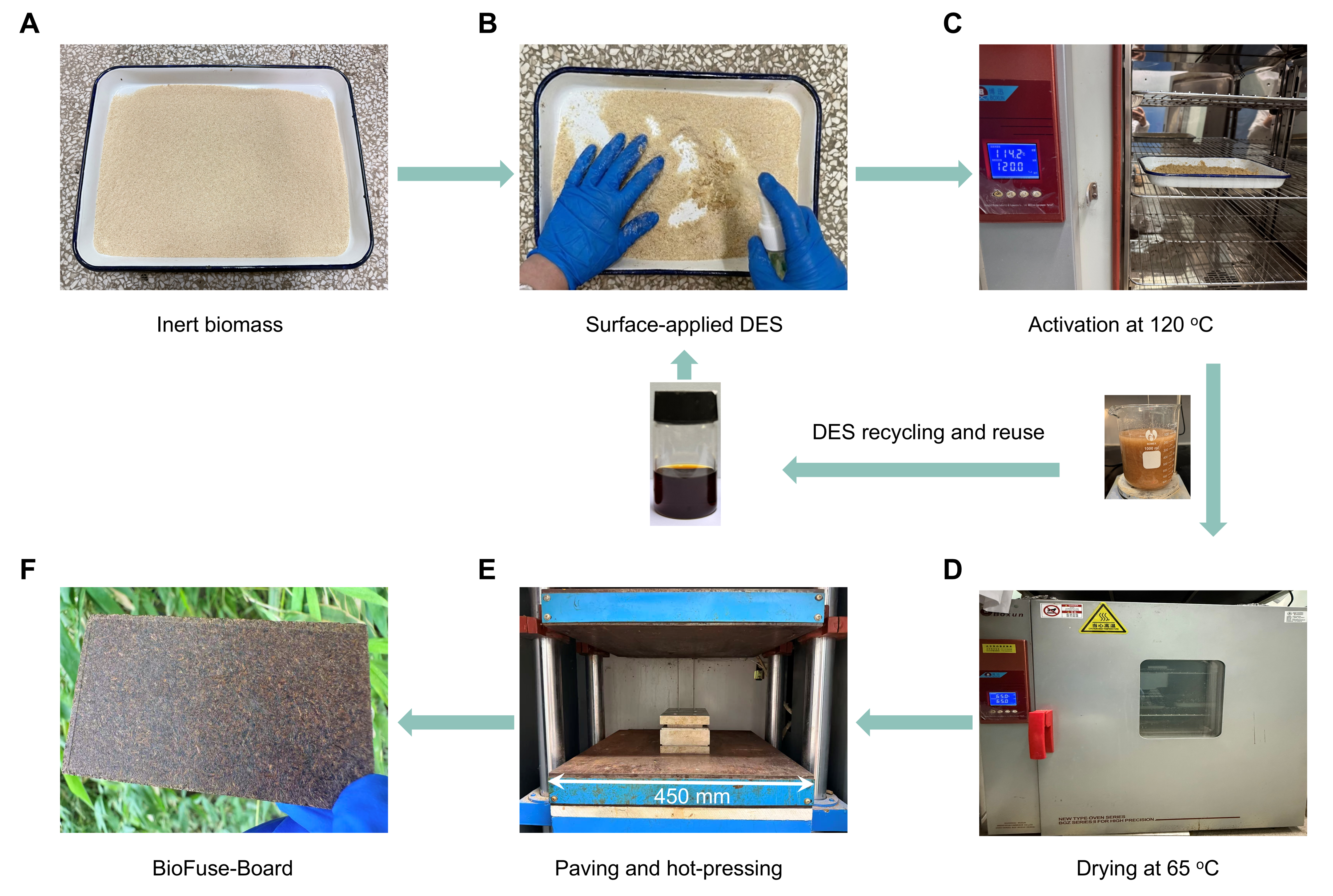
**

**Fig. S1.** **Laboratory-scale preparation of adhesive-free BioFuse-Board.** **(A)** Untreated lignocellulosic biomass feedstock. **(B)** Apply DES uniformly to the surface of lignocellulosic biomass feedstock. **(C)** Interfacial activation: Oven treatment at 120 ^o^C for 20 min. Add water to the surface of Ignited-Biomass to remove DES, enabling its recovery and recycling. **(D)** Drying the obtained Ignited-Biomass in an oven at 65 ^o^C adjusting the moisture content to 3%-5%. **(E)** Paving the activated biomass into a mat in the press mold and hot-pressing at 160 °C and 2 MPa for 30 min. **(F)** Resulting adhesive-free BioFuse-Board.

**
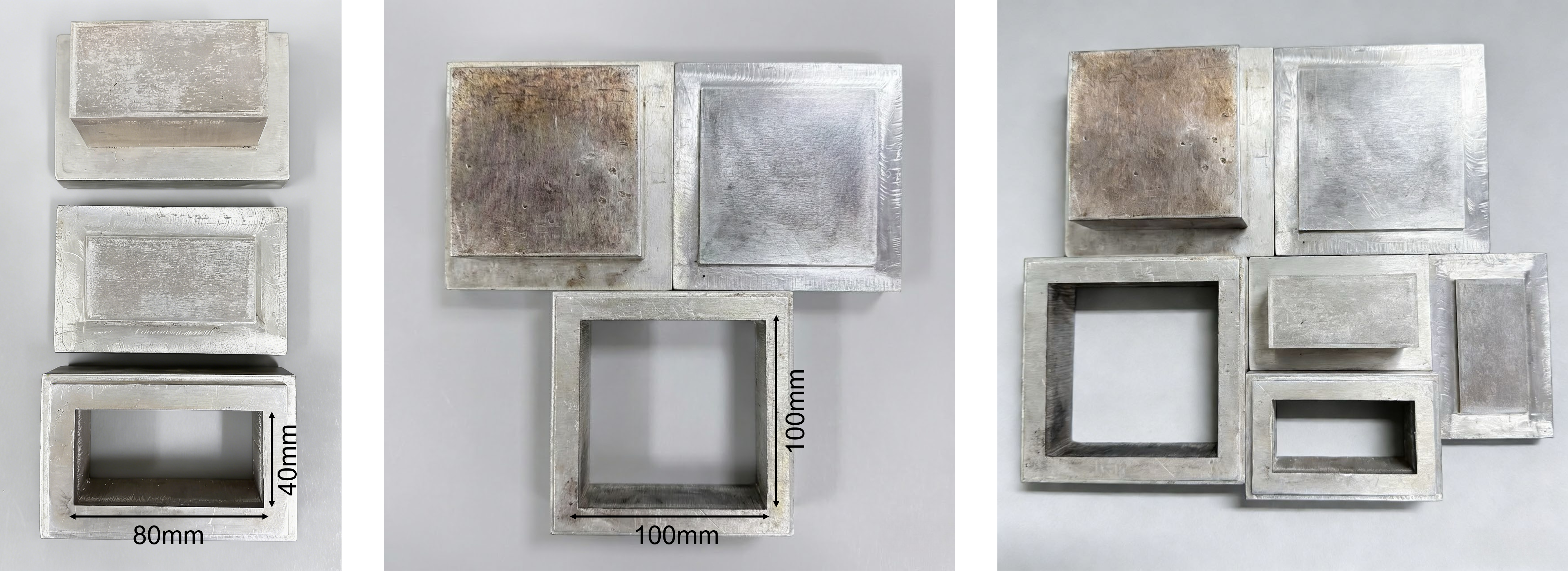
**

**Fig. S2.** **Photographs of molds.** Stainless-steel molds of different sizes used for board fabrication.


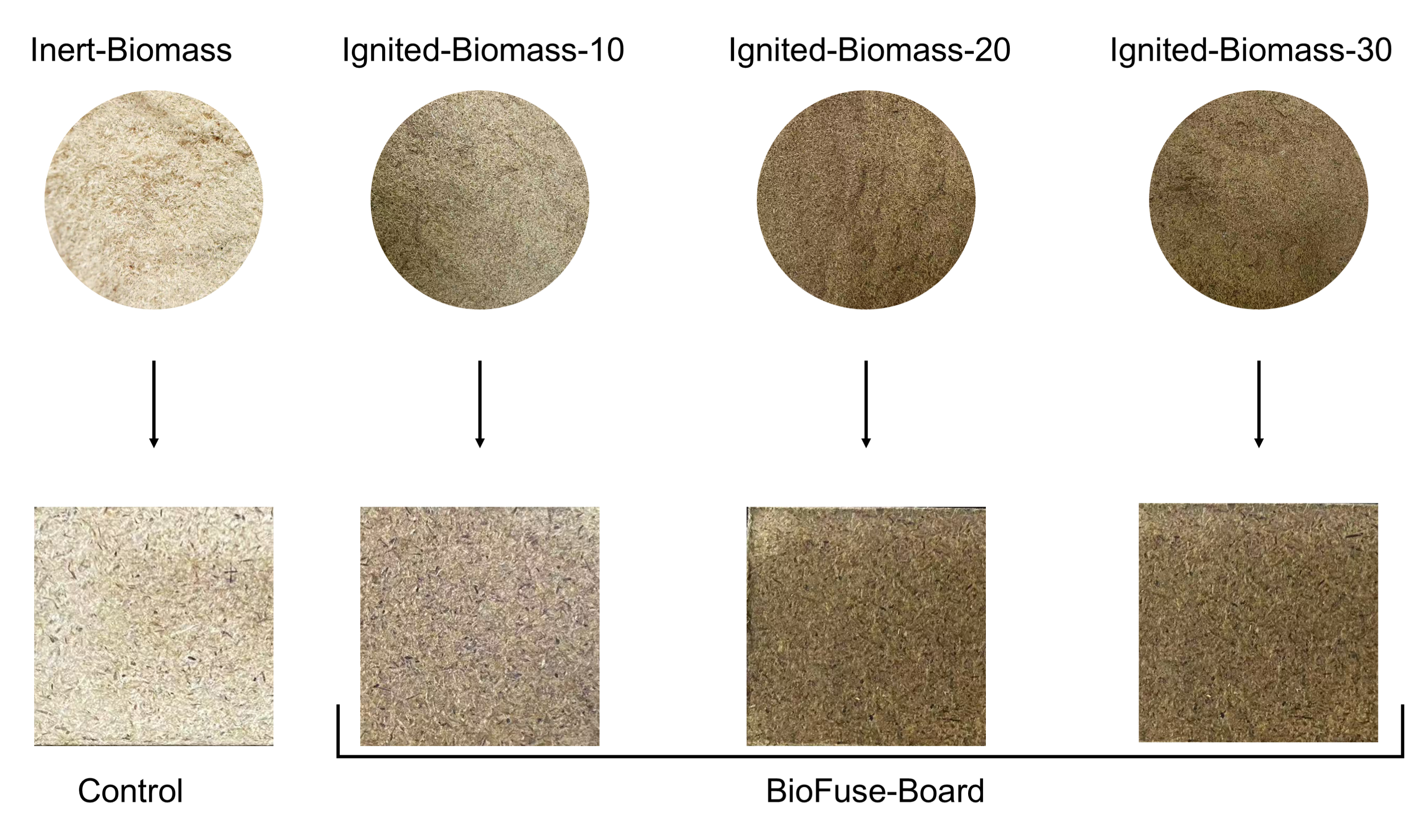


**Fig. S3. Visual evolution of furnish appearance during DES ignition at different times, consistent with progressive interfacial reprogramming.** Photographs show that the furnish gradually darkens from Inert-Biomass to Ignited-Biomass-10, -20, and -30, and the corresponding boards exhibit a similar darkening trend after hot pressing. This color evolution is consistent with progressive DES-induced interfacial activation and redistribution of lignin-rich components during ignition.

**
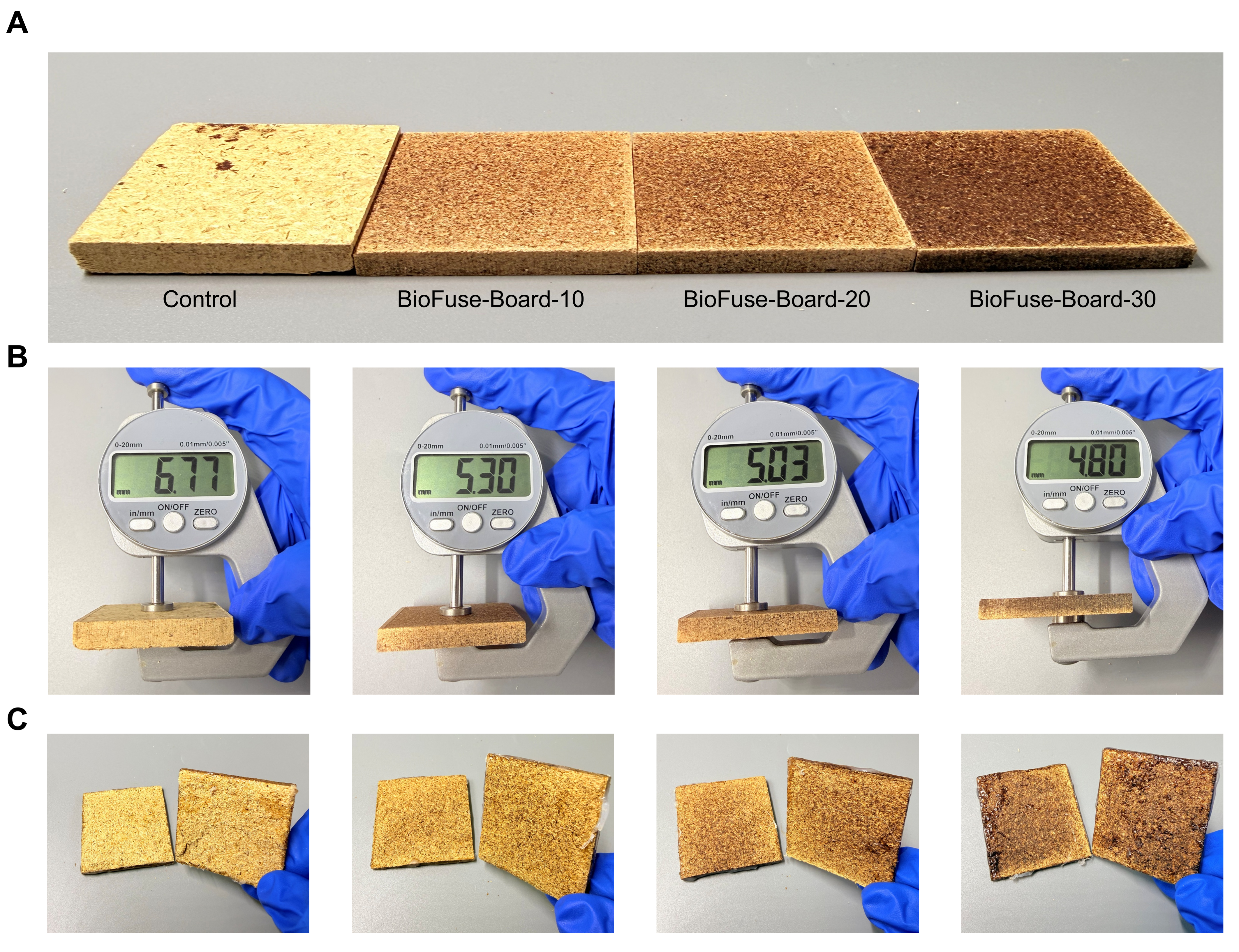
**

**Fig. S4.** **BioFuse-Board prepared at different DES activation time. (A)** Photographs of BioFuse-Board prepared from Ignited-Biomass with different DES activation time under the thermo-compression conditions of 160 °C, 2 MPa, and 30 min. **(B)** Photographs showing the thickness variation of BioFuse-Board prepared with different DES activation time indicate that as the activation time increases, the thickness of the boards gradually decreases. Specifically, the thickness of BioFuse-Board-20 is reduced by 1.74 mm compared to the Control (6.77 mm), suggesting that DES-ignited surface promote tighter interfacial bonding. **(C)** Fracture surface morphology of BioFuse-Board after internal bond strength testing with different DES activation time.

**
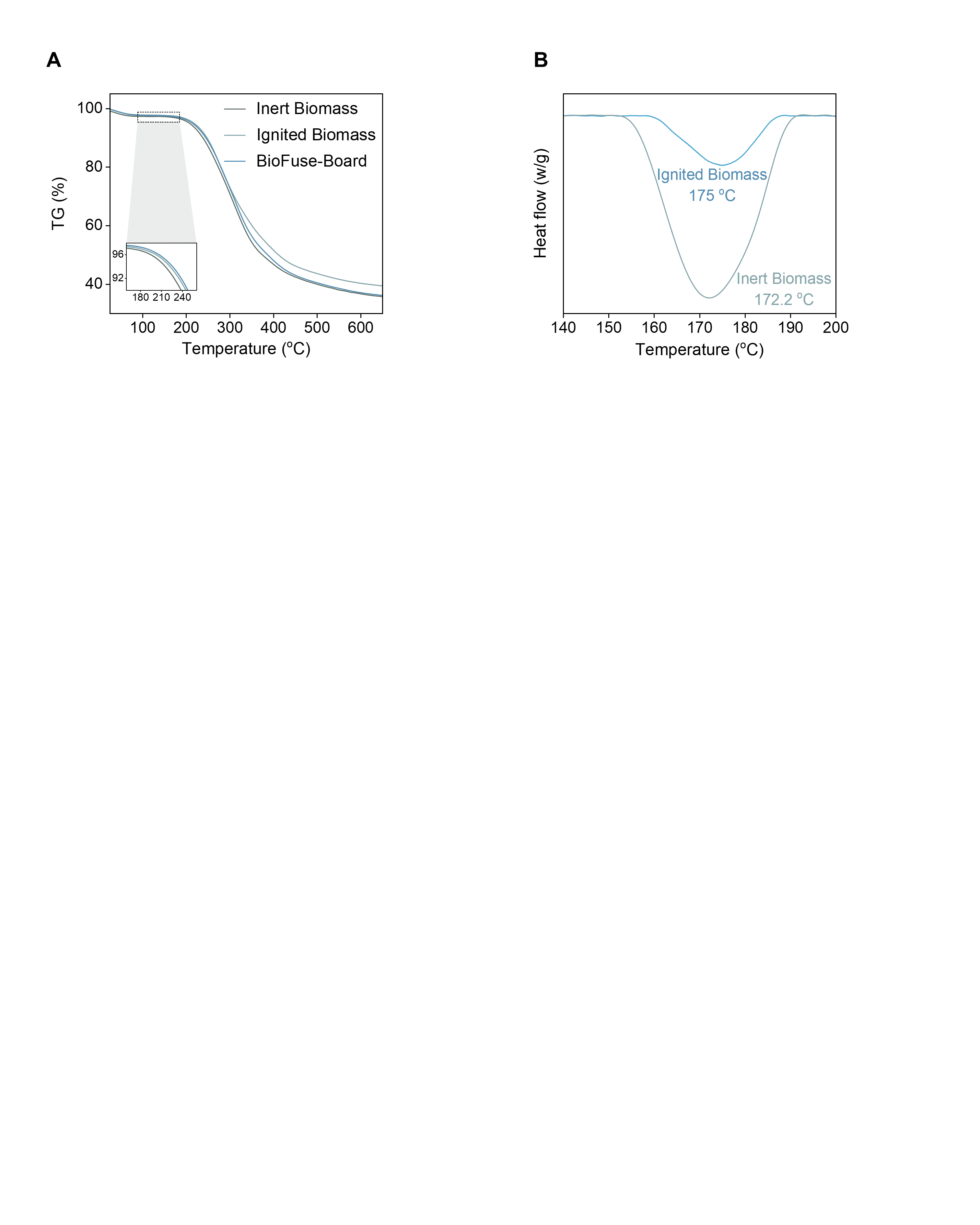
**

**Fig. S5. Thermal analysis of Inert-Biomass, Ignited-Biomass, and BioFuse-Board.**  (A) TGA curves showing that no substantial mass loss occurs at the selected DES ignition temperature of 120 °C or hot-pressing temperature of 160 °C. (B) DSC curves of Inert-Biomass and Ignited-Biomass, showing thermal-transition peaks at approximately 172.2 °C and 175 °C, respectively.

**Fig. S6. Comprehensive performance of our BioFuse-Board compared with commercial wood-based panels and reported lignocellulosic composites.** Internal bond strength versus thickness swelling comparing the BioFuse-Board developed in this work (star) with commercial wood-based panels and previously reported lignocellulosic bio-boards.

**
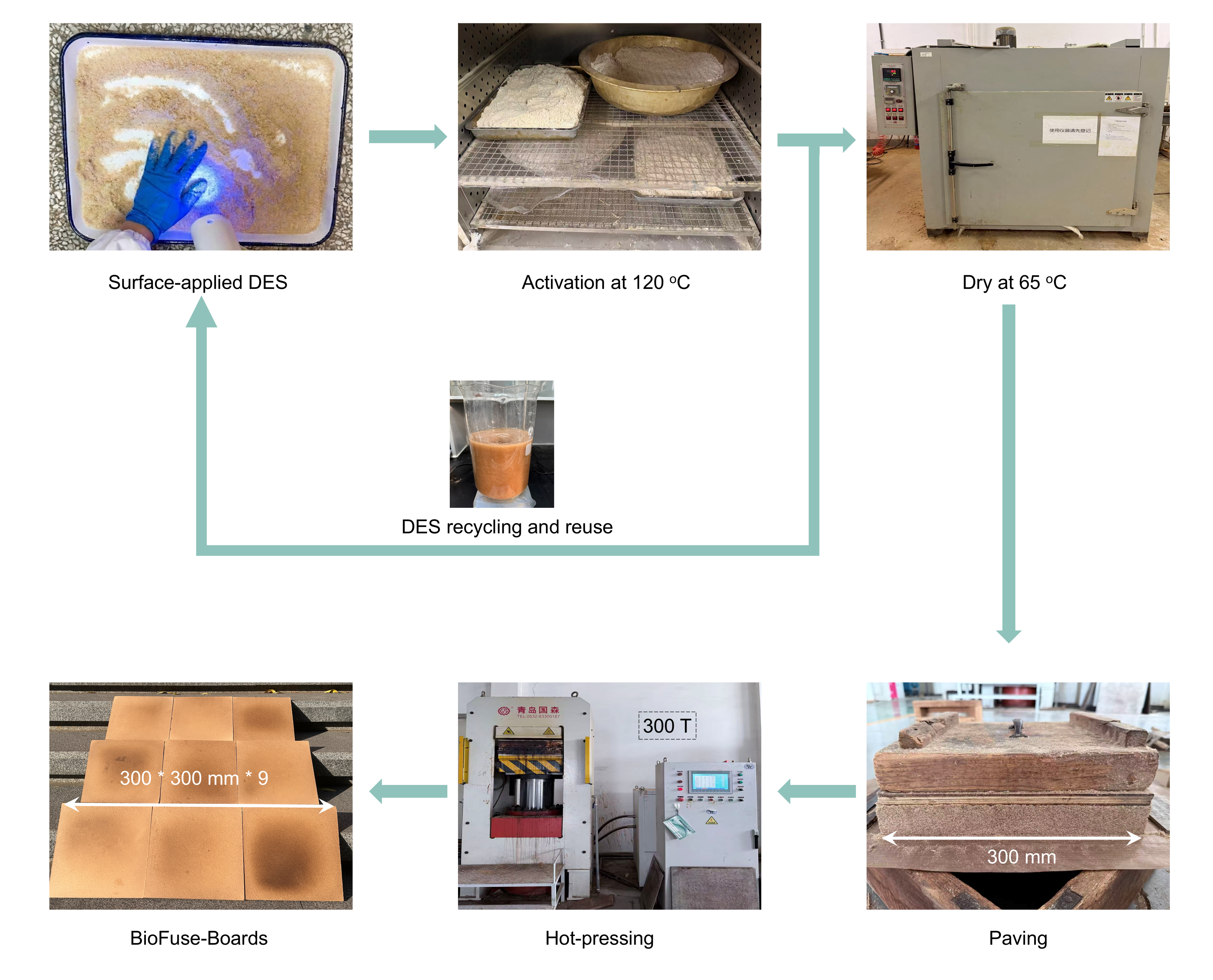
**

**Fig. S7. Pilot-scale preparation of BioFuse-Board.** Inert-Biomass is moistened with DES solvent and oven-heated to yield Ignited-Biomass. The activated material is paved into a 300 mm × 300 mm mold and hot-pressed in an industrial press (maximum pressure 20 MPa) to produce 300 mm × 300 mm BioFuse-Board. Despite the larger batch size and reduced heat-transfer efficiency at pilot scale, the Ignited-Biomass remains sufficiently plastic to form dense, defect-free boards, demonstrating robust process scalability.

**
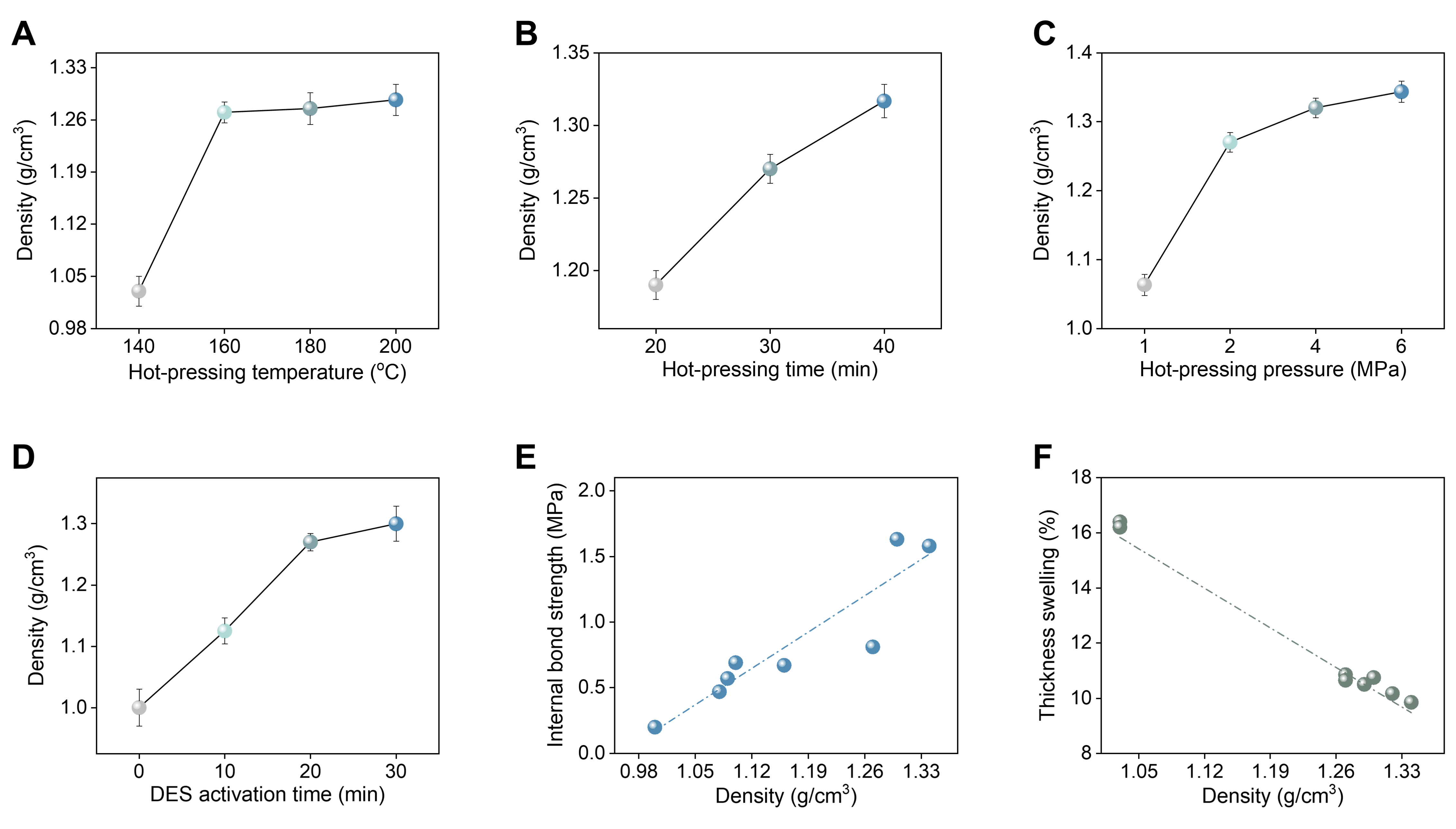
**

**Fig. S8. Control of BioFuse-board density and its link to performance. (A)** Density of board pressed with Ignited-biomass−20 showed an increase as a function of the hot-pressing temperature, under the condition of 2 MPa and 30 min. **(B)** Density of board pressed with Ignited-biomass−20 showed an increase as a function of the hot-pressing time, under the condition of 160 ^o^C and 2 MPa. **(C)** Density of board pressed with Ignited-biomass-20 showed an increase as a function of the hot-pressing pressure, under the condition of 160 ^o^C and 30 min. **(D)** Density of board pressed at 160 ^o^C and 2MPa increases with DES activation time from 0 to 30 min. **(E)** Internal bond strength plotted against density shows an approximately linear increase, indicating that higher densification improves cohesive strength. **(F)** Thickness swelling plotted against density shows a negative correlation, demonstrating that denser boards possess greater water resistance.

**
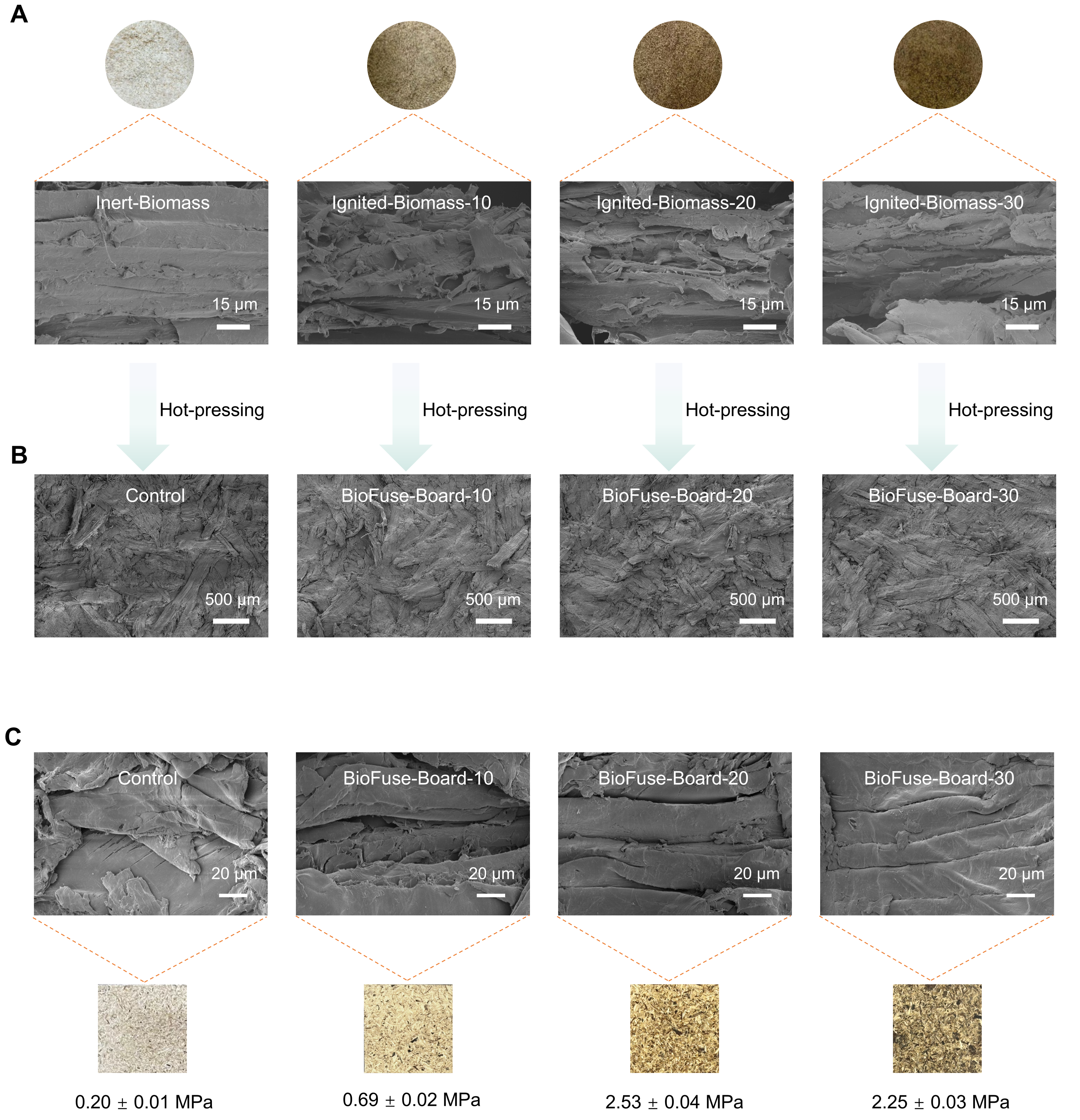
**

**Fig.S9. Evolution of cell-wall morphology with different DES ativacation time during ignition and fusion. (A)** SEM images of Imert-Biomass treated with different DES activation time show that as the activation time increases, the fiber surface becomes progressively rougher and more disordered, which facilitates mechanical interlocking and entanglement between fibers during the interfacial fusion process. However, excessive DES activation (Ignited-Biomass−30) also causes a certain degree of structural damage to the surface of the Ignited-Biomass, thereby leading to a decline in the physical and mechanical properties of the BioFuse-Board. **(B)** SEM images of the internal connection structure of BioFuse-Board prepared from Ignited-Biomass after different DES activation time at 160 °C and 2 MPa, reveal a state of mutual entanglement and interlocking among fibers, forming a progressively denser and more continuous internal structure. This trend is consistent with the gradual increase in the density of BioFuse‑Board as the DES activation time is extended. **(C)** Photographs and SEM images of BioFuse-Board show that as the DES activation time increases, the surface color gradually darkens, likely due to progressively more lignin migrating to the surface during ignition. SEM images display that BioFuse-Board with DES ignition have more extensively fused, interlocked cell-wall fragments and fewer open lumina, consistent with DES-induced cell-wall weakening and subsequent interfacial fusion during hot-pressing.

**
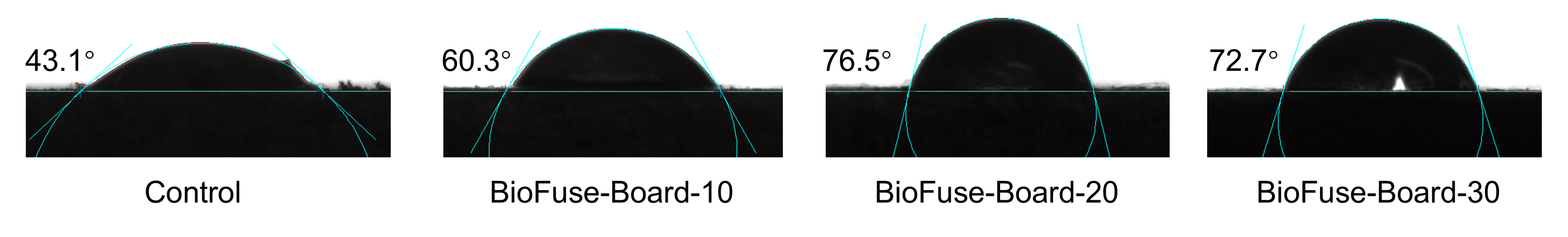
**

**Fig. S10.** **Contact angles of pure water after 25 seconds on the surface of BioFuse-Board.** Compression conditions: 160 ^o^C, 2 MPa, and 30 min. The increasing contact angels indicated that the DES ignition has a positive effect on enhancing the surficial hydrophobicity of samples. However, with longer pretreatment time (BioFuse-Board−30), excessive migration of lignin and hemicellulose to the surface compromised the structural integrity of the particles, resulting in a slight decline in the water resistance of the samples (as evidenced by a minor reduction in the contact angle).

**
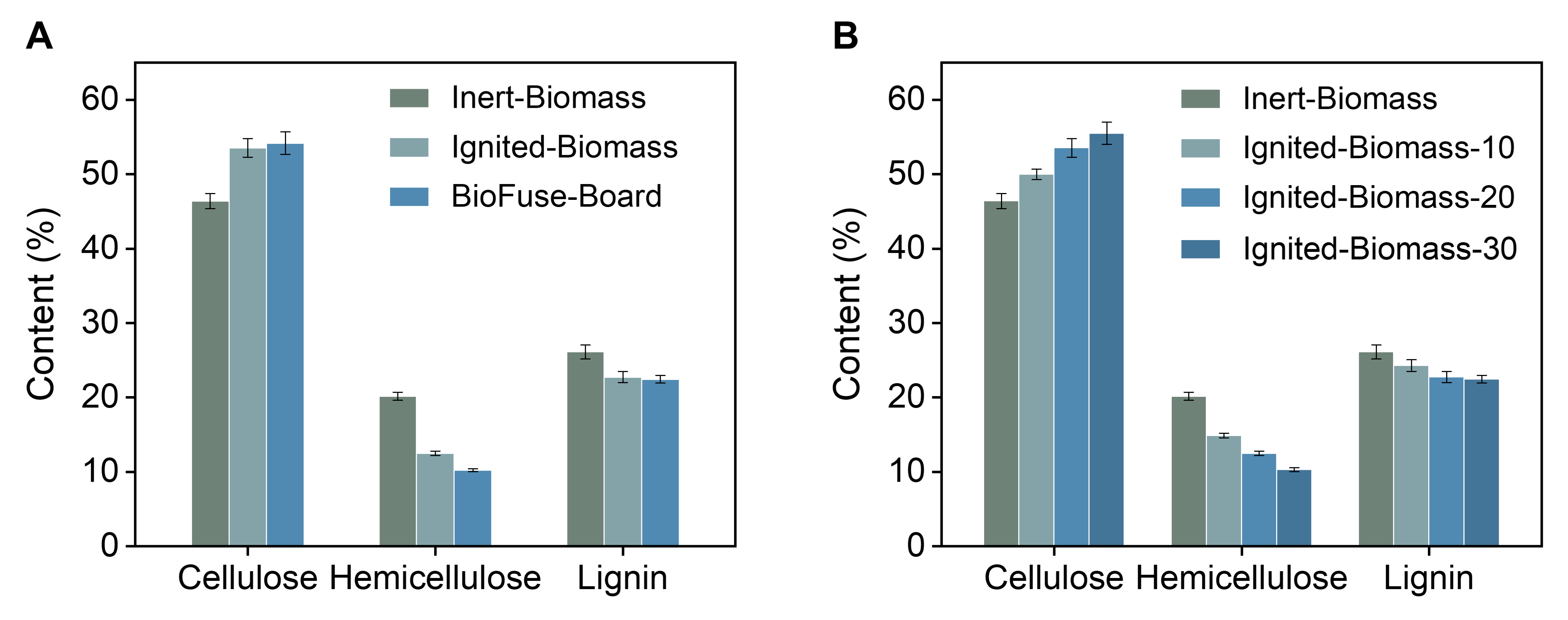
**

**Fig. S11. Changs in cellulose, hemicellulose and lignin contents during different activation time and fusion. (A)** Chemical composition analysis of Inert-Biomass, Ignited-Biomass, and the BioFuse-Board displays that DES ignition increases cellulose content and decreases hemicellulose. **(B)** With extended DES activation time, the cellulose content gradually increased in Ignited-Biomass, while the contents of hemicellulose and lignin decreased accordingly. This change facilitated the subsequent hot‑pressing fusion process. However, excessive migration of hemicellulose and lignin caused partial damage to the structural integrity of Ignited‑Biomass, which is consistent with the slight declines in mechanical properties and water‑resistant stability observed in boards fabricated from Ignited‑Biomass−30.

**
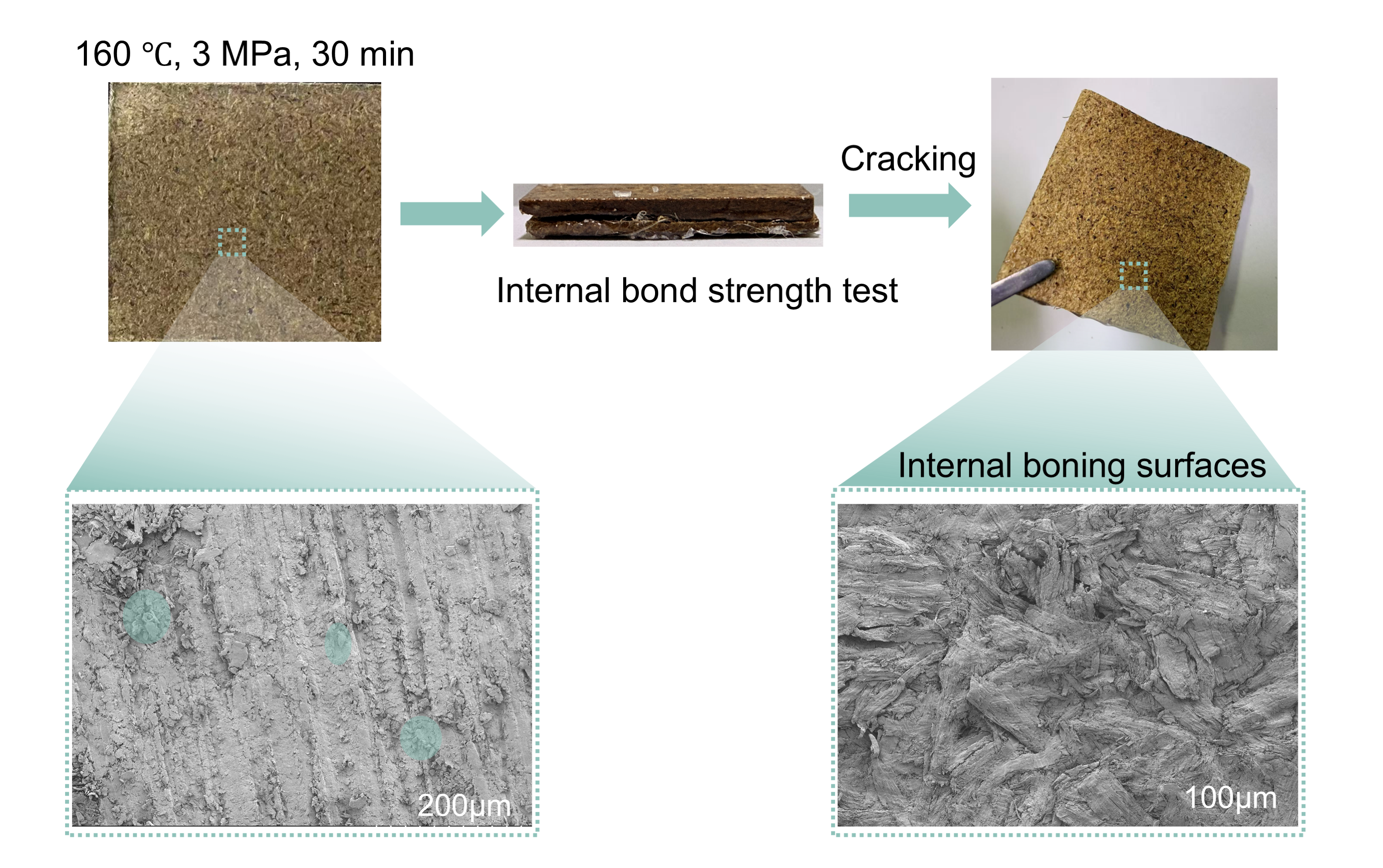
**

**Fig.S12. Fracture surface morphology of BioFuse-Board after internal bond strength testing.** Photographs and SEM images of internal facture surface after internal bond strength testing reveal a rough, fibrous morphology with extensive cohesive failure within the consolidated network and only limited interfacial gaps, consistent with strong fiber-fiber bonding induced by DES ignition.

**
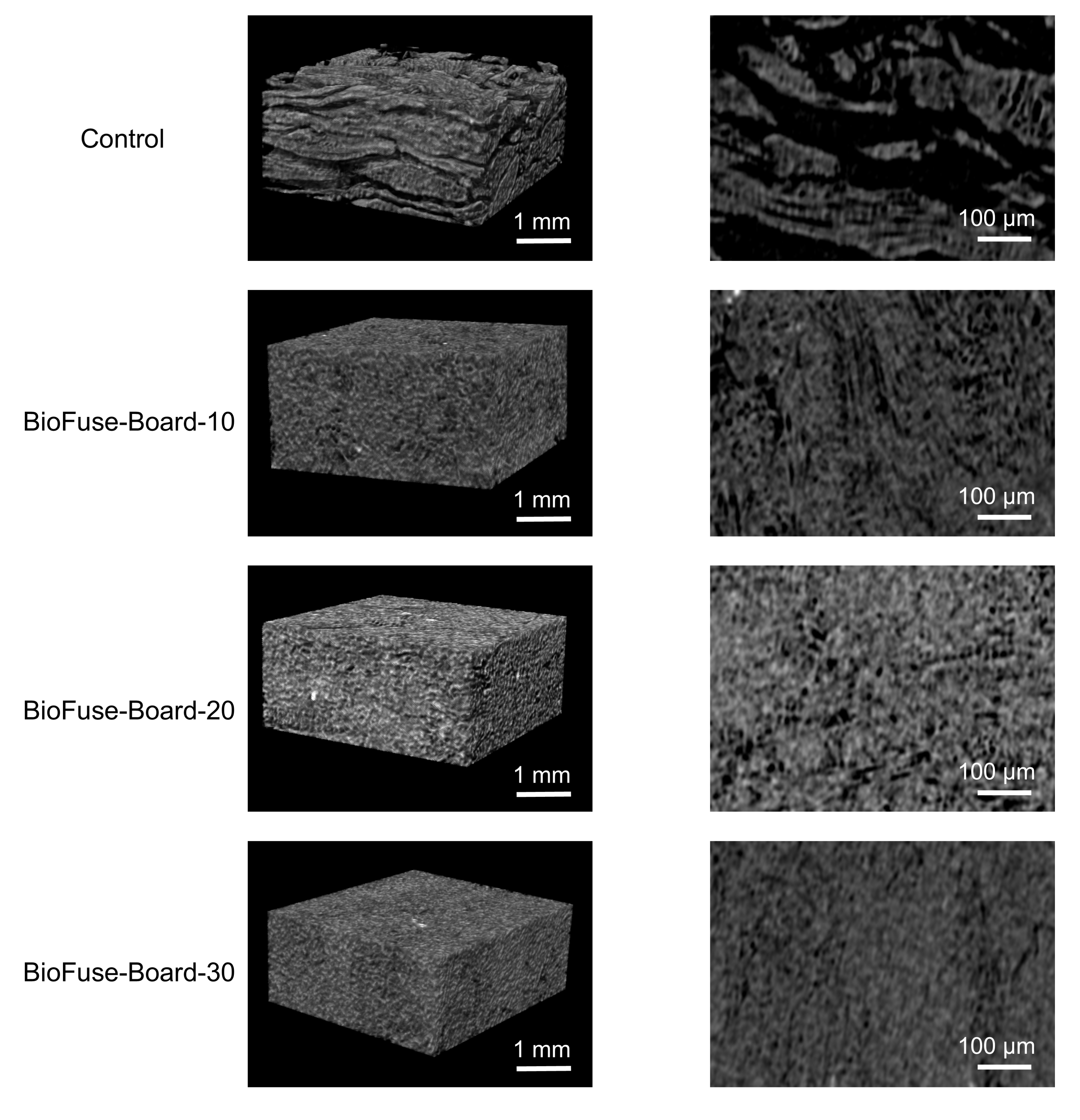
**

**Fig. S13. Three-dimensional micro-CT reconstruction of BioFuse-Board and non-DES-activated control samples.** The non-DES-activated sample displays a layered and porous architecture, while the BioFuse-Board exhibits a dense and homogeneous structure with minimal voids, attributable to effective interfacial fusion during the ignition process.

**
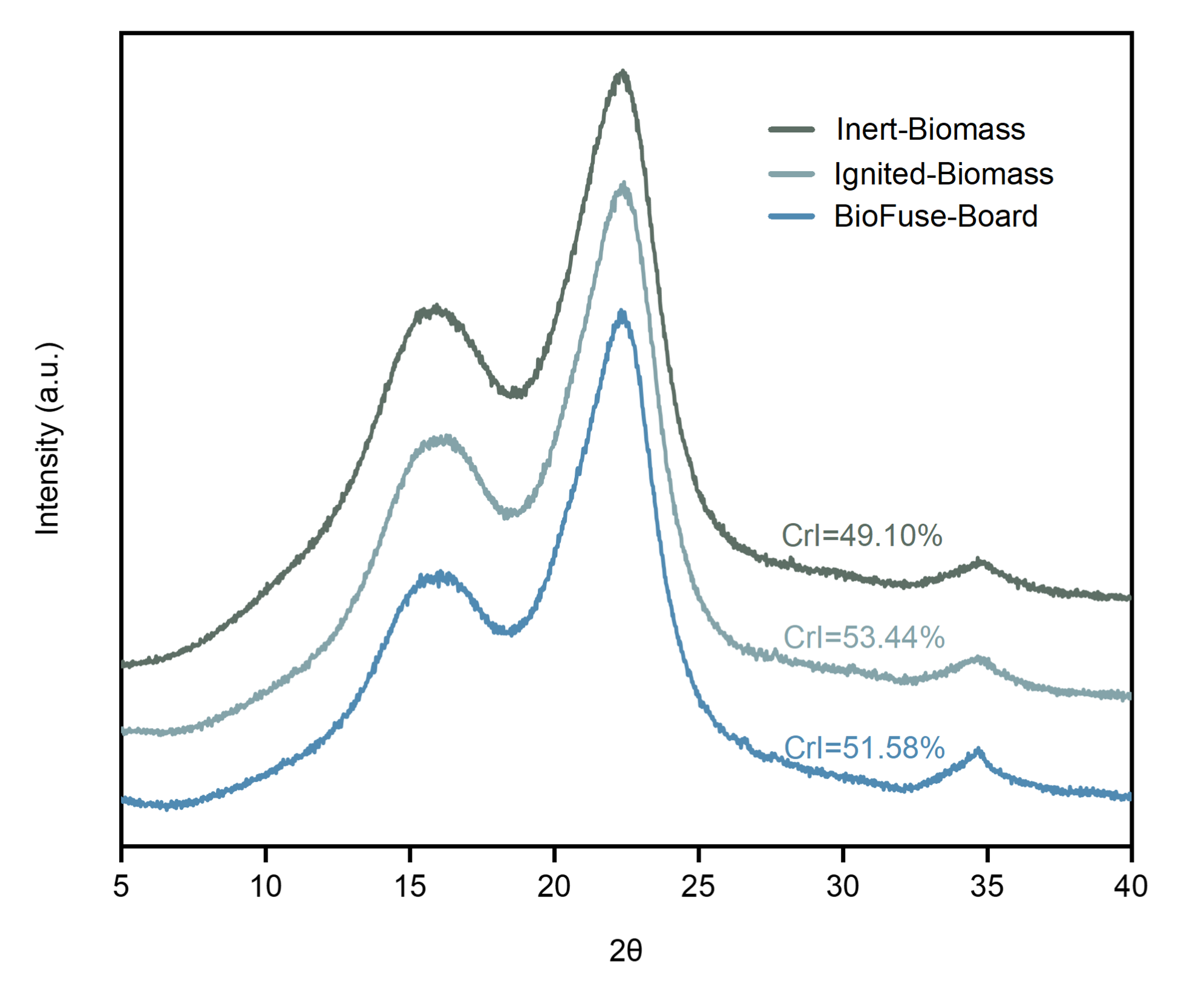
**

**Fig. S14. The XRD spectrogram of Inert-Biomass, Ignited-Biomass and the BioFuse-Board.** X-ray diffraction (XRD) patterns of the Inert-Biomass, Ignited-Biomass and the BioFuse-Board exhibited similar diffraction peaks (2θ = 18^o^ and 22.4^o^) indicated that the type of cellulose crystals in the fiber was not changed during the DES ignition and interfacial fusion. Meanwhile, the crystallinity index of the BioFuse-Board was 51.58%, showing a 2.48% increment compared to the Inert-Biomass (49.10%), which we attribute to the removal of hemicellulose and amorphous cellulose.

**
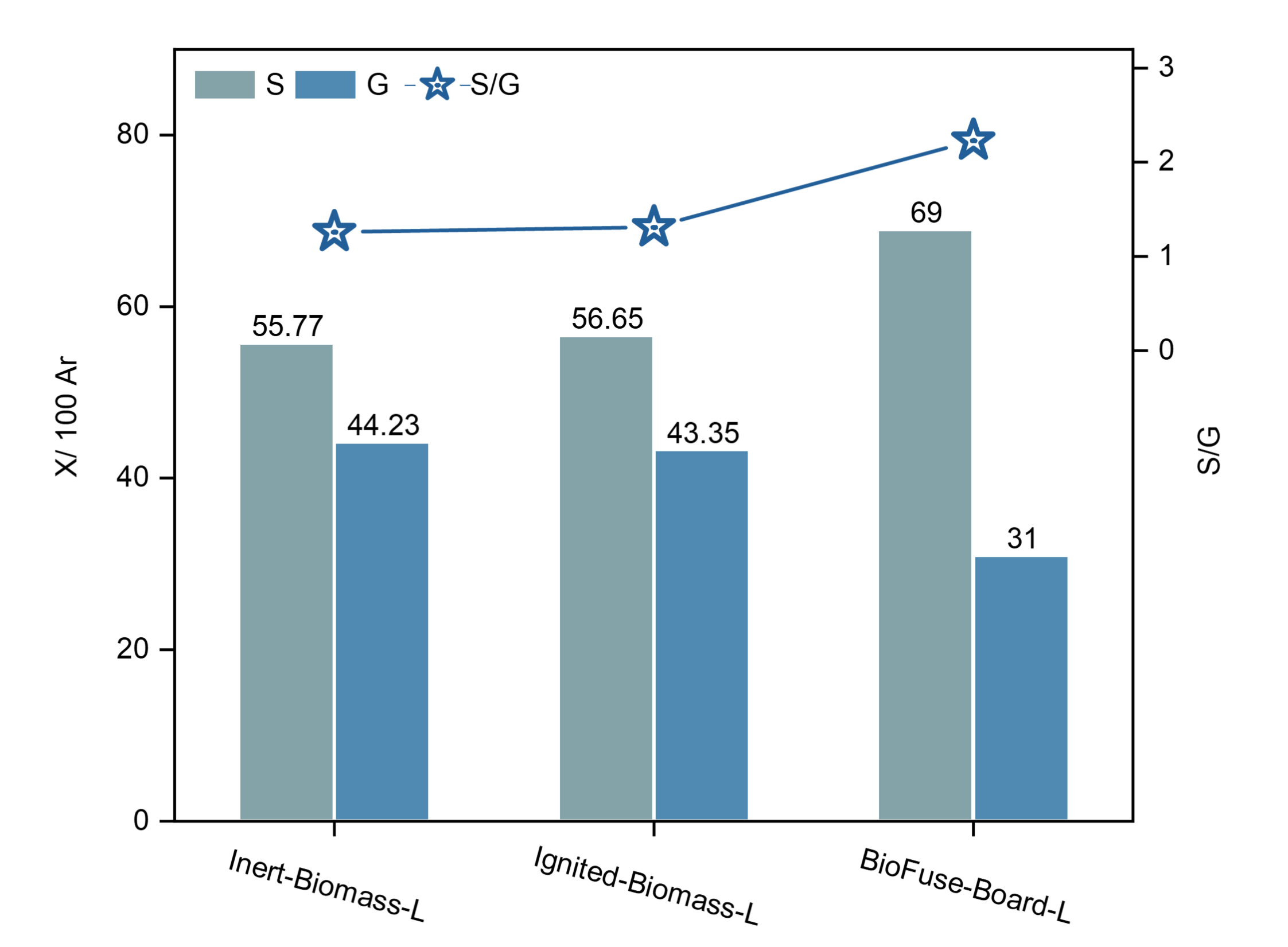
**

**Fig. S15. Changes in S- and G-type lignin units during ignition and fusion.** Quantification of S- and G-type structural units in lignin isolated from Inert-Biomass, Ignited-Biomass and the BioFuse-Board shows a progressive increase in S units and a decrease in G units, leading to a rising S/G ratio from 1.26 to 1.31 and finally 2.23, consistent with selective depolymerization and loss of G-type motifs during DES ignition followed by S-enriched recondensation during interfacial fusion.

**
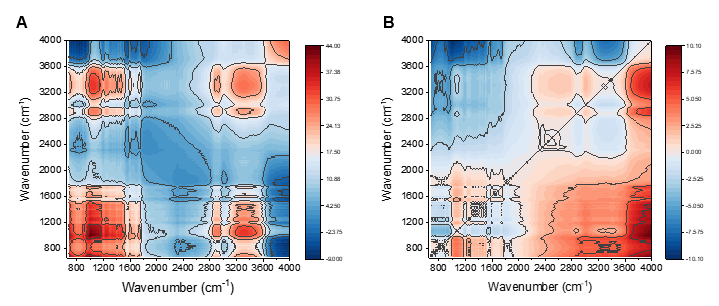
**

**Fig. S16. Two-dimensional correlation FTIR analysis during DES ignition and fusion. (A)** Synchronous 2D-FTIR spectrum showing correlated changes among O–H, carbonyl, aromatic lignin, and C–O/C–O–C regions, indicating coupled interfacial chemical evolution. **(B)** Asynchronous 2D-FTIR spectrum revealing sequential spectral responses, supporting a two-stage process from DES-induced interfacial activation to hot-pressing-enabled network locking.**
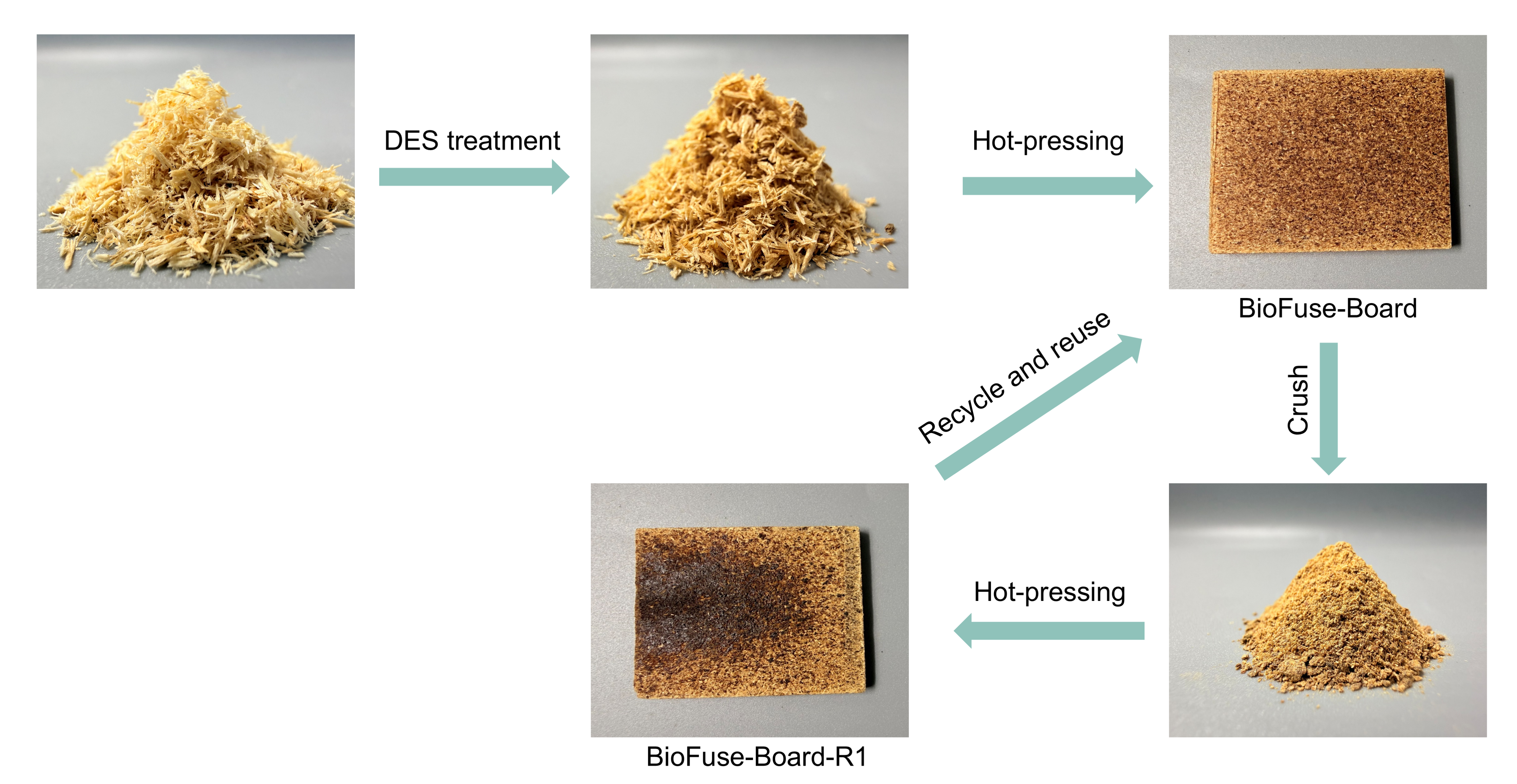
**

**Fig. S17.** **Closed-loop recycling of BioFuse-Board.** Closed-loop recycling process, in which end-of-life BioFuse-Boards are crushed and hot-pressed into new panels. End-of-life boards are pulverized to 60–100 mesh using a grinder, followed by wetting the Inert-Biomass surface with DES solution (1.5 times the mass of the wood power), and reactivating the Inert-Biomass by placing it in an oven at 120 ^o^C for 20 min, added distilled water to remove the DES from the Ignited-Biomass surface, adjusted to a moisture content of ~3%, and then hot-pressed at 160 °C and 2 MPa for 30 min to yield regenerated BioFuse-Board-R1.

**
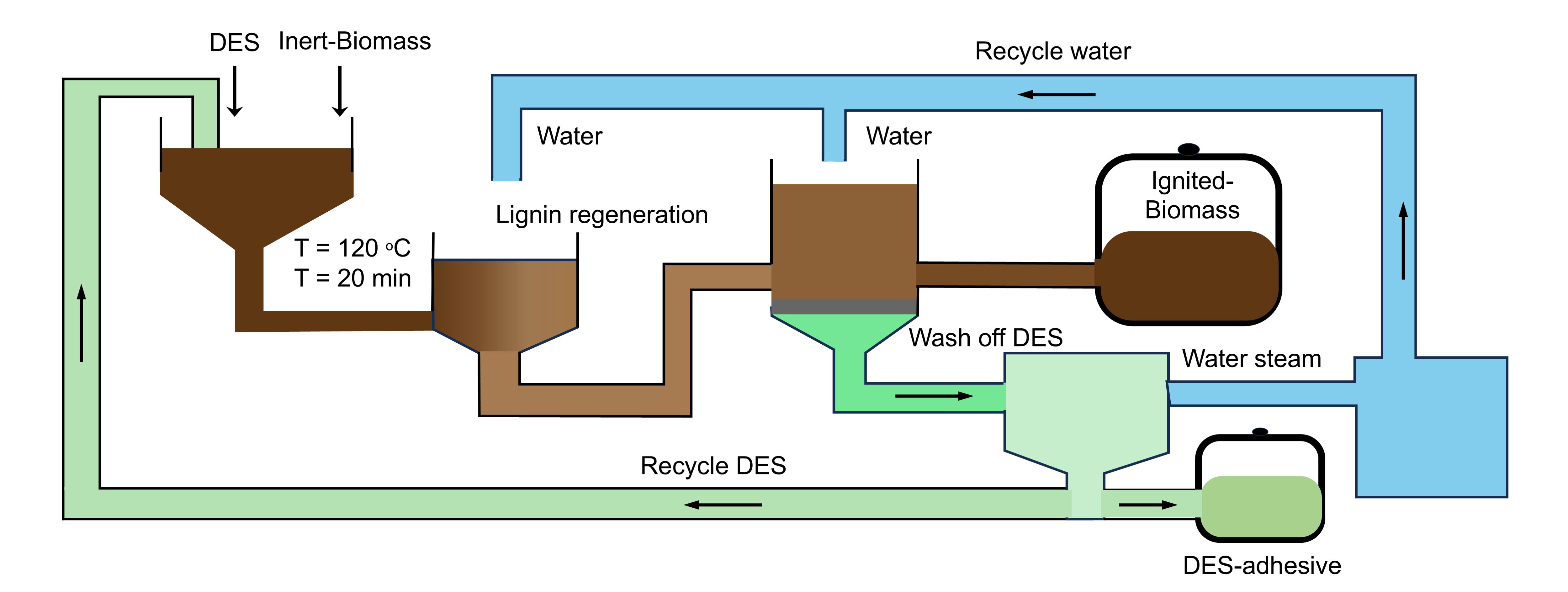
**

**Fig. S18. DES ignited Inert-Biomass interfacial process for preparing Ignited-Biomass and DES recycling flow chart.** After wetting the surface of Inert-Biomass with a DES solution and igniting it by heating at 120 °C for 20 min, water was added to remove the residual DES. The mixture was then filtered to separate Ignited-Biomass from the DES/water-containing filtrate. DES was subsequently recovered by heating the filtrate to evaporate water, and the recovered DES was reused to treat fresh Inert-Biomass. The evaporated water was collected via a condenser for reuse. After five reuse cycles, the spent DES solution was further used to prepare wood adhesives through a one-step heating method, contributing to a waste-minimized and discharge-reduced closed-loop process.

**Supplementary Tables S1 to S5**

**Table S1.** **Comparison of performance, processing strategy, and bonding mechanism of BioFuse-Board with representative adhesive-free and bio-based lignocellulosic board systems.**

| Biomass feedstock | Binder/  Polymer matrix (content) | Hot-pressing condition | Product type | Internal bonding strength (MPa) | 24-h thickness swelling (%) | Main bonding mechanism | Ref. |
| --- | --- | --- | --- | --- | --- | --- | --- |
| / | / | / | Commercial medium-density fiberboard (CN) | 0.60 | 20.0 | Self-bonding relies on fiber–fiber hydrogen bonding and CNF-assisted physical bridging. | [1] |
| / | / | / | Commercial particle board (CN) | 0.45 | 13.0 |  |  |
| Eucalyptus fibers | Melamine–formaldehyde–lignin resin (3%) | 1.0 MPa pre-press,  190 °C,  8.5 min | Fiberboard | 2.29 | 16.6 | Added co-modified lignin enhances bonding through chemical crosslinking and hydrogen bonding with wood fibers. | [2] |
| Poplar fibers | / | 170 °C,  8 MPa,  60 min | Structural materials | 2.24 | 14.8 | Bonding is driven by water-induced fiber plasticization, densification, hydrogen bonding, and localized H₂O₂ reinforcement. | [3] |
| Moso bamboo | / | 200 °C,  18.15 MPa,  13.2 min | Composites | 0.71 | 12.8 | Bamboo self-bonding arises from moisture/heat-induced component softening, interfacial interactions, and densification. | [4] |
| Poplar fibers | / | 190 °C,  10 min | Fiberboard | 0.85 | 14.1 | Citric acid promotes bonding through esterification crosslinking and lignin softening/re-curing. | [5] |
| Poplar fibers | / | 160 °C,  2 MPa,  30 min | BioFuse-Board | 2.53 | 10.0 | DES ignition–fusion bonds particles by in situ lignin/hemicellulose activation, redistribution, and hot-pressing-induced interfacial locking. | Our work |

**Table S2.** **Requirements for internal bond strength and 24-h thickness swelling of commercial wood-based panels in different standards.**

| No. | Density（g/cm^3^） | 24-h thickness swelling（%） | Internal bond strength （MPa） | Grade/application | Standard |
| --- | --- | --- | --- | --- | --- |
| 1 | 0.65~0.80 | 20.00 | 0.75 | Load-bearing fiberboard for high-humidity conditions | GB/T 11718-2021 Medium density fiberboard |
|  | / | 14.00 | 0.70 |  |  |
| 2 | ≥0.82 | ≤12.00 | ≥1.00 | Commercial Grade Ⅰ | GB/T 18102-2020 Laminate flooring |
|  | / | ≤17.00 |  | Household Grade Ⅰ |  |
| 3 | / | ≤17.00 | ≥0.80 | / | GB/T 12626.9-2015 Wet-process hardboard-Part 9, Load-bearing board for use in humid conditions |
|  |  | ≤15.00 |  |  |  |
| 4 | ≥0.8 | ≤8.00 | ≥0.80 | / | GB/T 15102-2017 Fiberboards and particleboards with decorative surface overlay of impregnated thermosetting resins |
| 5 | ＞0.8 | ≤12.00 | ≥1.20 | High-humidity-type high-density fiberboard | GB/T 31765-2015 High-density fiberboard |
|  |  | / | ≥0.95 | Ordinary high-density fiberboard |  |
|  |  |  | ≥0.90 |  |  |
|  |  | ≤16.00 | ≥1.20 | Moisture-resistant high-density fiberboard |  |
|  |  | ≤14.00 |  |  |  |
| 6 | / | ≤12.00 | ≥1.20 | / | LY/T 1611-2023 Fiberboard for floor substrate |
| 7 | ≥1.0 | ≤10.00 | ≥2.50 | Ordinary type | T/CTWPDA 11-2020 Ultra-high-density fiberboard |
|  |  | ≤8.00 | ≥2.50 | Indoor moisture-proof type |  |
|  |  | ≤5.00 | ≥3.00 | Indoor high moisture-proof type |  |
|  | ≥1.3 | ≤1.00 | ≥5.00 | Weather-resistant type |  |
| 8 | ≥0.8 | ≤30.00 | ≥1.20 | / | LY/T 3411-2024 Ultra-thin fiberboard |
| 9 | 0.5~1.0 | / | 0.30 | Grade 110 | ANSI A208-2022 Medium density fiberboard (MDF) for interior application |
|  |  |  | 1.05 | Grade 160 |  |
|  |  |  | 0.35 | Grade 210 |  |
|  |  |  | 1.50 | Grade 240 |  |
| 10 | ≥0.6 | ≤15.00 | ≥0.60 |  | EN 13986:2004 Wood-based panels for use in construction |
| 11 | / | ≤25.00 | ≥0.70 | Architectural high-density fiberboard for dry conditions | ISO 16895:2016 High-density fiberboard |
|  |  | ≤15.00 | ≥1.20 | High-density fiberboard for temperate and humid conditions |  |
|  |  | ≤13.00 | ≥0.70 | Architectural high-density fiberboard for environments with unrestricted humidity |  |
|  |  | ≤12.00 | ≥1.20 | High-density fiberboard used under high-humidity conditions |  |
| 12 | 1.2 | 10.00 | 2.53 | BioFuse-Board | Our work |

**Table. S3.** **Estimated raw-material cost for producing 1 ton of BioFuse-Board with Ignited-Biomass−20.**

| Raw material | Source | Price  (CNY t^−1^) | Usage  (t) | Cost  (CNY t^−1^ board) |
| --- | --- | --- | --- | --- |
| Poplar | Wood factory in Linyi City | ¥ 600 | 1.0 | ¥ 600 |
| Choline chloride (ChCl) | Macklin Chemical Co., Ltd | ¥ 15000 | 0.16 | ¥ 2400 |
| 1,4-butanediol  (1,4-BDO) |  | ¥ 8328 | 0.20 | ¥ 1665.6 |
| AlCl_3_ |  | ¥ 7800 | 0.006 | ¥ 46.8 |
| DES **^a^** | / | ¥ 31128 | 0.37/5 cycles | ¥ 822.48 |
| Industrial water | Local market | ¥ 9 | 0.3 | ¥ 2.7 |
| Electricity **^b^** | Local market | ¥ 0.5/kWh | 696.1 kWh | ¥ 348.05 |
| Total material cost | ¥ 1773.2 t^−1^ or $ 249.6 t^−1^ | | | |

**^a^** DES participated in 5 cycles.

**^b^** The energy includes the electricity used for heating, string, and vacuum filtration[6].

**Table. S4.** **XPS survey atomic composition and C1s-internal deconvolution results of Inert-**

**Biomass, Ignited-Biomass, and BioFuse-Board.**

| Sample | Surface C atomic % (survey) | Surface O atomic % (survey) | C/O ratio | C–C/C=C within C1s (%) | C–O within C1s  (%) | O–C=O within C1s  (%) |
| --- | --- | --- | --- | --- | --- | --- |
| Inert-Biomass | 72.49 | 27.51 | 2.64 | 51.2 | 38.3 | 10.6 |
| Ignited-Biomass | 65.59 | 34.41 | 1.91 | 41.3 | 46.1 | 12.6 |
| BioFuse-Board | 72.17 | 27.83 | 2.59 | 44.0 | 44.4 | 11.6 |

Survey C and O atomic percentages are referenced to the total XPS survey signal. C1s-internal proportions were calculated by renormalizing the three deconvoluted C1s sub-peak areas to 100% within each C1s envelope, reflecting the relative distribution of surface carbon chemical states.

**Table. S5.** **Comparison of techno-economic and environmental features of BioFuse-Board with conventional wood-based panels and related adhesive-free/bio-based board systems.**

| System | Chemical input | TEA / cost information | LCA information | recyclability | Ref. |
| --- | --- | --- | --- | --- | --- |
| Conventional panels | UF/MUF/PF/ resins | Mature low-cost industrial process | Fossil-derived binders; some systems have formaldehyde/VOC concerns | Limited remanufacturing due to cured thermoset networks | [7] |
| FWP | Lignin binder; MW-CNT filler; alkali treatment | No complete TEA reported | LCA lower than PE and PVC | Reuse potential mentioned; closed-loop board recycling not central | [8] |
| BBM | KOH/TEMPO oxidation; Ca²⁺ coordination | Energy use for 1 m³ BBM lower than plywood and carbon fiber composites | Lower GWP than plywood and carbon fiber composites | 84.1% bonding-strength retention after four recycling cycles | [9] |
| BioFuse-Board | Recoverable DES activation reagent | Raw-material cost: 1773.2 CNY t⁻¹ board; five-cycle DES cost: 822.48 CNY t⁻¹ board. | DES activation is the main environmental contributor; DES reuse and energy optimization are key | DES recovery, water reuse, spent-DES valorization, and board remanufacturing integrated | This work |

Direct comparison should be interpreted cautiously because functional units, system boundaries, production scales, energy mixes, and cost assumptions differ among studies.

**References** **and Notes**

1. D. Theng, G. Arbat, M. Delgado-Aguilar, F. Vilaseca, B. Ngo, P. Mutjé, All-lignocellulosic fiberboard from corn biomass and cellulose nanofibers. *Industrial Crops and Products* **76**, 166–173 (2015).

2. K. Zhang, Y. Liu, Z. Guo, J. Wang, Y. Liu, J. Zhao, P. Huo, Co-modification of corn straw lignin and its enhancement on glue-free fiberboard based on freezing activated wood fibers. *Industrial Crops and Products* **177**, 114452 (2022).

3. Q. Wang, L. Zhu, M. Wang, L. Cai, H. Ye, Z. Zhang, Y. Ren, Y. Yang, C. Chen, S. Ge, W. Gan, Development of eco-friendly and robust structural materials via binder-free lamination of waste biomass with help of finite element method. *Journal of Cleaner Production* **449**, 141715 (2024).

4. J. Shi, X. Xu, T. Zhong, W. Zhang, S. Yuan, X. Feng, R. Sang, C. Xia, H. Chen, B. Fei, Fabrication and Application of Eco-friendly Bamboo Self-Bonded Composites for Furniture. *ACS Sustainable Chemistry & Engineering* **11**, 7833–7843 (2023).

5. K. Jiang, J. Wang, D. Fan, F. Chu, Y. Chen, Citric Acid-Mediated Lignocellulosic Fiber Bonding for Sustainable Materials: Multiscale Insights into Molecular Cross-Linking and Physical Interlocking. *ACS Sustainable Chemistry & Engineering* **13**, 21568–21578 (2025).

6. Q. Xia, C. Chen, Y. Yao, J. Li, S. He, Y. Zhou, T. Li, X. Pan, Y. Yao, L. Hu, A strong, biodegradable and recyclable lignocellulosic bioplastic. *Nature Sustainability* **4**, 627–635 (2021).

7. I. Calvez, R. Garcia, A. Koubaa, V. Landry, A. Cloutier, Recent Advances in Bio-Based Adhesives and Formaldehyde-Free Technologies for Wood-Based Panel Manufacturing. *Current Forestry Reports* **10**, 386–400 (2024).

8. Y. Yang, L. Zhang, S. Ge, H. Huo, K. Huang, M. Rezakazemi, Z. Zhang, Sustainable wood fiber composites with antistatic and high-thermal-conductivity performance for energy-efficient home environments. *Journal of Materials Chemistry A* **13**, 15075–15087 (2025).

9. Z. Fu, K. Wang, X. Yu, W. Liu, W. Lao, W. Hong, Y. Zhang, W. Yu, Y. Lu, Synergistic interaction of non-covalent and coordination bond to fabricate recyclable bamboo-based material. *The Innovation Materials* **3**, 100132 (2025).
